# Rubber plantations are impermeable to an avian understory specialist in Sri Lanka

**DOI:** 10.1186/s40462-024-00484-8

**Published:** 2024-06-17

**Authors:** Salindra K. Dayananda, Harsha F. Athukorala, Indika Peabotuwage, Chandralal Kumara, Tharindu Ranasinghe, Dhammithra Samarasinghe, Ana Gouveia, Sarath W. Kotagama, Christos Mammides, Aiwu Jiang, Eben Goodale

**Affiliations:** 1 Guangxi Key Laboratory of Forest Ecology and Conservation, College of Forestry, Guangxi University, Nanning, 530004 Guangxi China; 2 Institute of Environment and Ecology, Tsinghua Shenzhen International Graduate School, Tsinghua University, Shenzhen, 518055 Guangdong China; 3 Field Ornithology Group of Sri Lanka, Department of Zoology and Environment Science, Faculty of Science, University of Colombo, Colombo, 00700 Western Province Sri Lanka; 4 Wild Island Foundation, 6A, Mendis Lane, Moratuwa, 10400 Western Province Sri Lanka; 5 Nature Conservation Unit, Frederick University, 1036 Pallouriotisa, Nicosia, Cyprus; 6 Department of Health and Environmental Science, Xi’an Jiaotong-Liverpool University, Suzhou, 215123 Jiangsu China

**Keywords:** Agriculture, Bird movements, Understory birds, Habitat connectivity, Landscape ecology, Radio telemetry, Translocations, Sri Lanka

## Abstract

**Background:**

Understanding how landscape characteristics affect animal movement is essential for conservation in human-dominated habitats. A fundamental question is how monoculture agroforests, including rubber and tea plantations, affect wildlife and its movement. Experimental translocations represent an important technique to assess animals’ habitat selection while moving through agricultural matrices, especially when complemented with observations of birds’ natural movements, and with “control” translocations, in which birds are moved within their natural habitat such as forest. Yet, experimental translocations have been little used for birds outside the Western Hemisphere.

**Methods:**

We conducted experimental translocations and home-range measurements on an understory forest specialist, Brown-capped Babbler (BCBA, *Pellorneum fuscocapillus*), and a forest generalist, Tickell’s Blue Flycatcher (TBFL, *Cyornis tickelliae*). These species were studied in three rubber plantations, which also included some open areas mostly planted with tea, and in three forest reserves of Sri Lanka.

**Results:**

Four of the five BCBAs translocated within disturbed habitats (rubber plantations) could not return to their capture locations. However, all four individuals within undisturbed habitats (forest reserves) successfully returned to their point of origin within 10.5 daytime hours. In contrast, all TBFLs returned to their capture locations in both disturbed (n = 7) and undisturbed habitats (n = 3) within 11.3 daytime hours. A Cox-proportional survival model demonstrated that the percentage of rubber cover decreased return time, similar to the effect of open-area cover. The home range surveys (n = 13 for BCBA, n = 10 for TBFL) revealed that very little of the birds’ natural home-ranges was covered by rubber (0.2% for BCBA, 13.1% for TBFL at 50% Kernel Density Estimates KDE). Home range size for BCBA was approximately half the size in disturbed habitats compared to undisturbed ones, although there was no significant difference between habitats for TBFL.

**Conclusions:**

We conclude that rubber plantations can be impermeable to understory habitat specialist birds, and even generalist species may avoid them long-term. Our findings highlight the potential utility of strips of native vegetation, particularly those featuring understory layers, as corridors to facilitate the movement of forest specialists in landscapes dominated by rubber plantations and other types of disturbed habitats.

**Supplementary Information:**

The online version contains supplementary material available at 10.1186/s40462-024-00484-8.

## Introduction

Land-use change due to agricultural intensification is a major cause of the biodiversity crisis. Agriculture has expanded in the biodiversity-rich tropics over the past half century, and further expansion is expected in the coming century [55, 68]. The issue extends beyond habitat loss to include fragmentation, and establishment of modified human-dominated matrices that surround increasingly smaller forest fragments. These matrices restrict gene flow, create inhospitable elements to wildlife, and provide few poor resources while imposing a high risk of predation [24, 58]. Because of these changes, landscapes will experience declines in species richness of forest specialists [44, 46], diminished functional connectivity [9, 61], and interference with ecosystem services [45]. However, current scientific understanding of how forest floral and faunal populations respond to these rapid ecological changes remains inadequate.

Beyond understanding how land-use change affects the presence of biodiversity, it is both timely and crucial to comprehend how these alterations affect animal movement, especially in the context of rapidly changing landscapes [11, 31, 33]. Apart from its necessity in acquiring basic ecological needs, such as finding food and shelter, movement is critical to maintaining populations [25] and thereby avoiding extinctions [26], through colonizing new meta-populations [22], and keeping genetically viable populations through continuous gene flow [10, 41, 43]. It is particularly important to understand how agricultural matrices act as barriers against animal movement, especially in tropical fragmented landscapes, but the methodologies employed to investigate this complex question are not yet fully developed.

One well-repeated and established experimental methodology to measure functional connectivity is experimental translocations, which have often been performed on birds [15]. The translocation paradigm involves capturing a bird from its established territory, releasing it into an unfamiliar habitat (presumably outside its territory), and subsequently observing its homing behavior or navigation through available landscape matrices [4, 20]. Although translocation methods offer valuable insights into adult dispersal patterns, they have been criticized for not adequately capturing juvenile or natal dispersal, a significant dimension of animal movement [50]. The artificial nature of these experiments has also been criticized since it simulates homing rather than dispersal. Further, the experiments can be confounded by methodological inconsistencies and the stress the experiments exert on the animals [6]. To mitigate these limitations and provide more robust conclusions, Betts et al. [6] suggested the incorporation of control experiments in which birds are translocated within their natural habitat. This enables a clearer understanding of the influence of habitat, independent of the translocation method itself [6]. Despite the weaknesses discussed above, experimental translocations are considered an important tool to measure functional connectivity, as they standardize animals’ motivation to move [6, 15].

Among tropical crops, monoculture rubber plantations play a key role in altering landscapes, especially in Asia [65]. Monoculture rubber plantations have direct impacts on mammals [48], birds [69], amphibians [3], ants [42], termites [28], and soil micro- and mesofauna biodiversity [35, 54]. Yet how rubber plantations affect animal movement is unclear, because although they are generally resource-poor [57], they are an agroforest that has a fairly continuous canopy. Having a closed canopy may make forest animals less likely to perceive rubber plantations as risky, as compared to crops grown in open areas.

Here we aimed to compare the permeability of monoculture rubber plantations to that of tea and other types of open areas, using experimental translocations and home range measurements of Sri Lankan birds. We selected two species: the Brown-capped Babbler (BCBA, *Pellorneum fuscocapillu*s), an endemic forest specialist that prefers the understory of mature forests, and the Tickell's Blue Flycatcher (TBFL, *Cyornis tickelliae*), a forest generalist that prefers the mid-canopy and canopy strata, and demonstrates a marked preference for forested habitats, yet exhibits ecological flexibility concerning the types of forest it can occupy. We hypothesized that BCBA would encounter significant challenges in navigating through monoculture rubber plantations and open habitats following translocation events. In contrast, we anticipated that TBFL would exhibit greater ease in traversing human-disturbed areas, at least in moving through rubber monoculture landscapes. Additionally, we measured birds' home ranges within these diverse habitats to discern their unmanipulated habitat preferences, as well as to examine variations in home-range size and body condition in relation to human disturbance. We hypothesized that BCBA would avoid rubber monocultures in their territories and that individuals of both species would manifest reduced territorial expanses and suboptimal body conditions when situated within human-disturbed habitats.

## Methods

### Study area

**Fig. 1:**
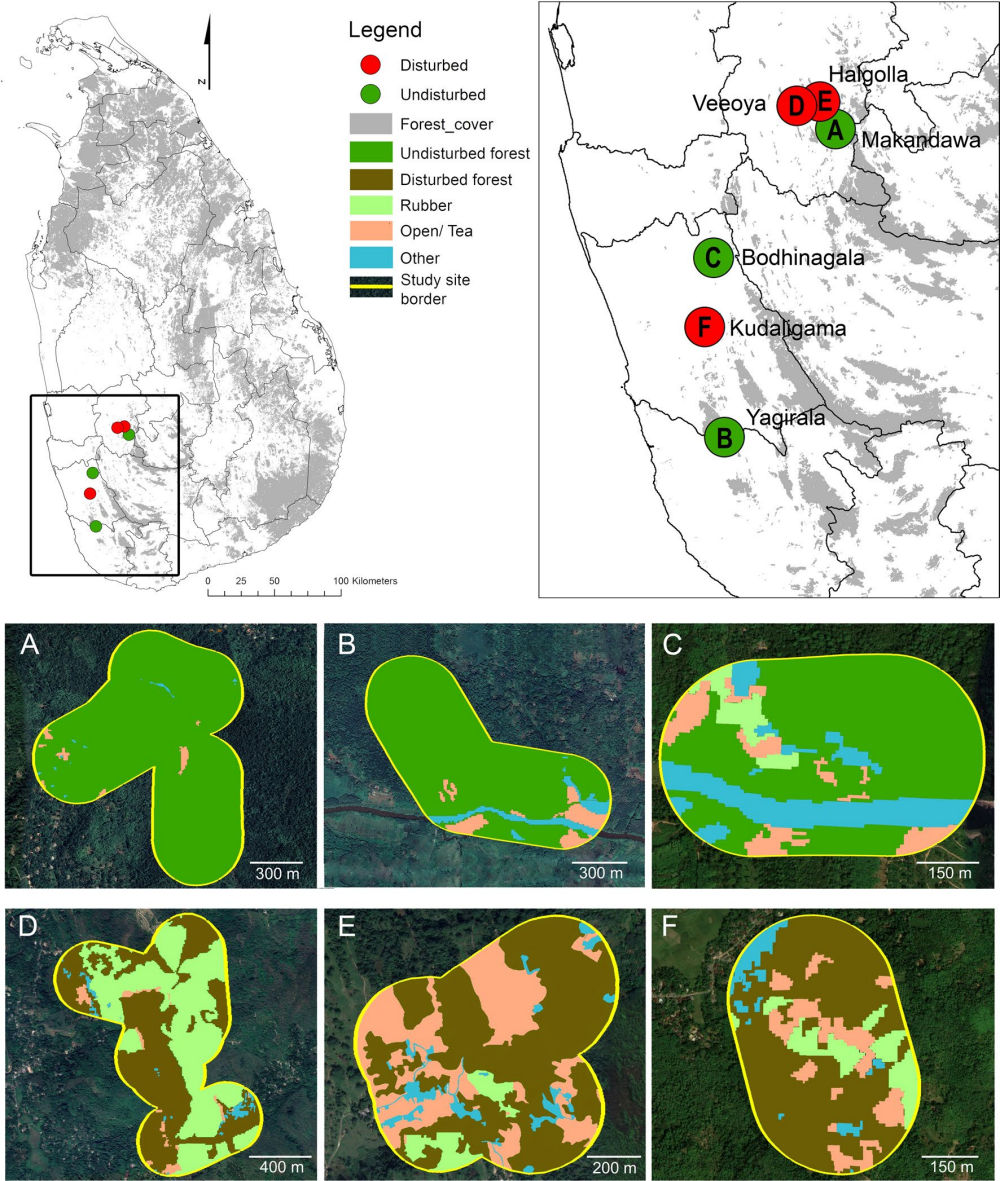
Map of the study sites, including the three forest reserves and the three rubber plantations (the circles representing these locations in the upper panels are not to-scale). In the imagery for each site towards the bottom of the figure, land-use types are shown for any areas that fell within ellipses during a translocation experiment or were part of a home range (**A** = Makandawa Conservation Forest, **B** = Yagirala Forest Reserve, **C** = Bodhinagala Forest Reserve, **D** = Veeoya Estate, **E** = Halgolla Estate, and **F** = Kudaligama Estate)

Field experiments were conducted in six study sites in the lowland southwestern wet zone of Sri Lanka (Fig. 1). Three sites were privately owned rubber plantations: Halgolla Estate (7.0421 N, 80.3682 N, ~ 550 m asl; 1196.2 ha), Kudaligama Estate (6.5863 N, 80.1369 E, ~ 110 m asl; 5.5 ha), and Veeoya Estate (7.0333 N, 80.3232 E, ~ 135 m asl; 951 ha). We also worked in forest reserves, including Bodhinagala Forest Reserve (6.7275 N, 80.1592 E, ~ 105 m asl; 282.6 ha), Makandawa Conservation Forest (6.9888 N, 80.4031 E, 121 m asl; 1155 ha), and Yagirala Forest Reserve (6.3647 N, 80.1760 E, ~ 60 m asl; 2390 ha). The minimum distance between sites was 5.26 km (between Veeoya and Halgolla). The mean annual temperature in this region is 28 °C, and the mean annual rainfall is ~ 2000 mm, with two wet seasons (from May to September and November to February) due to monsoon rains.

In general, this region is characterized by an agricultural mosaic with scattered forest fragments [52]. Most of the remaining large forest patches (area > 100 ha) have been designated as protected reserves and harbor tropical wet evergreen forests [21]. Human activities are prohibited inside these reserves, and we refer to them hereafter as undisturbed habitats. The rubber plantations included a mosaic of land-uses including monoculture rubber, some small tea estates (< 10 ha), some patches of abandoned agriculture now containing secondary vegetation, and forest fragments degraded by human activities (e.g., firewood collection, low-intensity logging). These rubber plantations were situated in the landscape among other plantations with monoculture rubber, and small towns with well-wooded home gardens. Hereafter we refer to these habitats as (human) disturbed.

### Survey methods

#### Species selection

BCBA and TBFL are both listed as Least Concern by the IUCN, and are highly territorial and relatively abundant insectivorous species, with different micro-habitat preferences and flight abilities. BCBA (~ 30.0 g) is an endemic species predominantly inhabiting the forest understory. Characterized by limited flying capabilities, this species typically lives in male–female pairs [37]. Although BCBA is known to persist in dense scrubs, overgrown land near villages, and disturbed secondary undergrowth in the buffer zones around forested areas [14], we classified BCBA as a forest specialist due to its extremely low probability of occurrence within non-forested habitats. TBFL (~ 15 g) is a strong-flying flycatcher most often found in the forest subcanopy and uses other strata opportunistically. It is also typically found in male–female pairs. The species exhibits habitat flexibility, occupying a diverse range of environments from forests and well-wooded home gardens to the peripheries of agricultural fields [13]. Accordingly, we categorized TBFL as a forest generalist.

We sampled the species in disturbed and undisturbed habitats for both translocation experiments and home range measurements (Table 1); only male individuals were sampled. A total of 19 BCBAs and 17 TBFLs were studied, of which nine BCBAs and 10 TBFLs were translocated. Among the translocations, three BCBAs and four TBFLs were translocated inside the forest, in what we consider a “control experiment”. Two BCBAs, which dropped the transmitters, and one TBFL, which escaped before transmitter attachment, were considered as involved in mistrials of translocations and were not tracked (nor counted in Table 1). Thirteen BCBAs and 10 TBFLs were monitored for home ranges. Five BCBAs and four TBFLs were subjected to both translocations and home range surveys, with home range surveys completed after the translocations.

**Table 1:** A summary of the sampling for the translocation experiments and home range surveys for the two bird species

	Undisturbed	Disturbed	Total
*Brown-capped Babbler (BCBA)*			
Translocations	3	6	9
Home range surveys	5 + (3)	3 + (2)	8 + (5) = 13
Total	8	9	17
*Tickell’s Blue Flycatcher (TBFL)*			
Translocations	4	6	10
Home range surveys	2 + (1)	4 + (3)	6 + (4) = 10
Total	6	10	16

Some individuals of each species were subjected to both translocations and home range surveys, which are indicated within parentheses

#### Experimental translocations

All the translocations were conducted during the non-breeding season (July-February, in the years between 2019 and 2022). The peak breeding season for BCBA is reported to be from March to May, and for TBFL, it is between March and June [13, 14]. Thus, sampling avoided these periods, and in addition, all the individuals were carefully observed for cloacal protuberance and other behaviors indicating active breeding (e.g., collecting nesting materials, nest building); birds showing breeding activity were not subjected to translocation experiments. All individuals were captured in forested habitats, either in the forest reserves or in small forest fragments situated within the plantations (n = 9, mean size = 9.64 ha, range from 1 to 23 ha; two in Halgolla Estate, four in Kudaligama Estate, and three in Veeoya Estate). Birds were attracted to ground-based mist nets opened from ground level up to 6 m in height, using territorial song playbacks. Once captured, each bird was marked with a colour ring for individual identification and basic biometric data was collected (body weight, wing length, length of the first secondary feather, etc.), with such processing time taking on average 8.2 ± 3.5 min (for this value, and following measurements of variability, we report standard deviation).

Each individual subjected to a translocation experiment was fitted with a 0.75 g radio transmitter (Holohil Systems Ltd: Type BD-2) with a lifespan of 4 weeks. A harness made of cotton thread (0.25 mm, 8 ply) was used on TBFL, which decayed after four weeks. For BCBA, we used nylon thread (0.4 mm) because the first bird pecked on the cotton thread until it broke. Therefore, all the BCBA individuals translocated (both individuals which successfully homed or not) were recaptured to remove the nylon thread and the radio tag. Radio tag attachment time averaged 26.5 ± 11.7 min. Soon after tag attachment, individuals were checked for any signs of stress (un-sustained flapping, partially closed eyes, fluffed plumage) [4] or injury; but none of the individuals manipulated showed any of these symptoms. After tag attachment, individuals were placed in cotton bags and transported by field vehicle or foot (in control treatments) to a unique release location. During transportation, extra attention was paid to minimize the stress by driving slowly and when walking by minimizing vibration; transportation time averaged 16.9 ± 12.3 min. The total time during the entire process from capture to release, including processing time, tagging time, and transportation time, averaged 51.0 ± 22.2 min. All handling protocols were approved by the ethical review committee of the Institute of Biology, Sri Lanka (ERC IOBSL 195 06 2019).

Release locations were selected from Google Earth Pro and then ground-truthed (i.e. verified in the field) before conducting any experiments. Because we assumed that the released birds would perform homing movements, release locations in disturbed habitats were selected such that a straight line between capture and release locations intersected at least one matrix land-use type (either rubber or open area); we tried to select release locations where birds would have the choice between rubber and open area matrices. All the individuals captured in the PAs were translocated within the same forest in the control experiments and released outside their territory to reduce site familiarity, as it may confound the results [6]. Each combination of capture and release locations was used once for each individual, but multiple translocations were done at the same landscape for individuals of different species. The mean translocation distance was 393 ± 126.6 m (see Appendix 1, included in Supplementary Information). We were advised to keep the maximum translocation distance < 500 m by the National Forestry Sector Research Committee of the Department of Forest Conservation of Sri Lanka to minimize the chance that any birds would have long-term consequences from the experimental procedure.

All the release locations were comprised of three or more native or introduced plants forming a bushy patch of very small size, ranging from 6 to 45 m^2^ (average = 16.9 ± 11.9 m^2^). This size provided immediate shelter for the bird yet was too small for establishing a territory (all home ranges were larger than 1 ha). Once we reached the release site, the bird was kept still for one minute to reduce stress due to transportation, and then released. It was monitored continuously for 10 min to ensure it could perform sustained flight between branches (for TBFL) or was able to hop on the ground and make short flights (for BCBA). None of the individuals displayed abnormal movement, and all soon performed normal behaviors such as feeding, calling and preening. All the individuals were released before 14:00 h (mean release time = 9:50 h).

Upon release, birds were followed by two teams, each comprising two experienced observers with a signal receiver and three-element YAGI antennae (TRX-1000s, Wildlife Materials, Inc.), handheld global positioning system unit (GPSmap 62s/64s, Garmin), a range finder and a binocular. To increase the accuracy of positions, locations were recorded once every 15 min using a simultaneous bi-angulation technique, wherein both teams, located more than 50 m from each other, took fixed signal bearings simultaneously [34]. Tracking was continued from 6:00 to 18:30 h each day until the bird returned to its capture site. Individuals who failed to return home and did not orient towards capture locations were recaptured and released to their capture site after the third or fourth day after translocation (in practice, this involved only BCBAs).

#### Home range surveys

Home range surveys were conducted from November 2019 to July 2021. To survey the home ranges, we marked 13 (n = 8 for undisturbed habitats and n = 5 for disturbed habitats) BCBAs and 10 (n = 3 for undisturbed habitats and n = 7 for disturbed habitats) TBFLs. As in the translocations, all birds were males, and we avoided collecting observations of nesting birds. Two observers monitored each individual for three or four consecutive days during daylight hours (from 06:00 to 18:45). Restrictions on our sampling due to the COVID-19 pandemic made some differences between sampling; however, 17 of 23 surveys were done across multiple visits to the home range in both the non-breeding and the breeding season when birds were not actively nesting (Supplementary Table 1). The size of home ranges measured during a single visit of four consecutive days (n = 4) were within the range of those observed in more than one visit (n = 19).

A subset of individuals, comprising five BCBAs and four TBFLs, had previously undergone translocation procedures. We conducted a Mann Whitney U-test, due to non-normality and low sample size, to compare the home ranges of these translocated individuals against those not subjected to translocations, for both species separately. Our analyses yielded no evidence to suggest that the translocation process affected home range size for either species (for BCBA: W = 27, P = 0.35 and for TBFL: W = 14, P = 0.76). During home range monitoring, observed points were recorded at 15-min intervals to maintain consistency with the observational framework utilized in the translocation studies. Acoustic playback techniques were employed sparingly using a Bluetooth speaker (song tracks were from our own recordings and three tracks downloaded from www.xeno-canto.org) and used only when an individual's location remained undetermined following a 10-min observational period. We were careful not to perform playback when we were potentially near to the edge of the home range.

#### Land-use survey and classification

All the bi-angulated bearings were fed to LOAS software (Ecological Software Solutions, Urnash, Switzerland) to obtain the location fixes. Fix accuracy was confirmed by comparing actual observed points (n = 10) with estimated locations by the LOAS software and was found to have 4.1 ± 1.3 m and 5.2 ± 3.6 m error for estates and forests, respectively. In total, 715 locations were recorded for BCBA and 262 for TBFL, with 46.7% and 41.6% visually confirmed for the two species, respectively.

To demarcate an area for land-use type analysis, we drew a straight line between the capture and release locations and constructed an ellipsoid around that, following Tremblay and St. Clair [60]. The maximum perpendicular deviation from the straight line to bird locations was 235 m for TBFL and 80 m for BCBA; thus, we set 250 m as the maximum width of the ellipsoid. ArcMap 10.8 was used to overlay a grid of 10 × 10 m cells on SPOT satellite images of the sites (made in January 2021), and the grid cells in the ellipsoid were then demarcated into the following four land-use types: ‘forested’, ‘rubber’, ‘open area’ and ‘built-up area’. More than 80% of the grid cells in disturbed habitats were ground-truthed by observers while conducting translocations and home range surveys; ellipsoids in PAs were ground-truthed only for non-forest land-use types.

### Statistical analyses

#### Homing time and success

All statistical analyses were done in R version 4.0.3 (R Development Core Team 2020). We considered return time as the response variable and used a Cox-proportional hazards model to determine the effect of explanatory variables using the ‘survival’ package [59]. Cox regression is suitable to model bird movements with ‘time to event’ data (here, return time to capture location) and also considers whether an event occurred within the observed period (here, return success within 4 days). The first step was making a Kaplan–Meier survival graph for the two species in the two habitats, with birds that did not return (all BCBA) right-censored. Differences between these four curves were assessed using the semi-parametric log-rank test and the multiple comparisons were Bonferroni-corrected.

Subsequently, we constructed a more complex multivariate model to examine the effects of land-use cover. The explanatory variables examined in this model included the percentage cover of forest, rubber and open-area (the percentage of the built-up area being negligible), along with translocation distance, species and treatment type (control or not). Initially, we checked the collinearity among the explanatory variables, ensuring that Pearson’s correlation coefficient between each variable was < 0.6 and the variance inflation factors were < 2. Since treatment had a high variance inflation factor (> 4.0) and forest cover was negatively correlated with rubber cover (− 0.87, P < 0.001), the final model included only species, translocation distance, rubber cover, and open-area cover. Additionally, the full model encompassed all conceivable 2-way interaction terms between species and the various cover variables. Variables or interactions that exhibited no statistical significance (P > 0.05) were systematically eliminated from the full model in a sequential manner. This process continued until the most parsimonious model, containing only statistically significant predictors, was identified. We constructed the survival model in the ‘coxme’ package, which allowed the incorporation of a random factor to account for variation attributable to the study sites.

#### Home range and body condition analysis

All birds showed strong site fidelity, did not make linear movements, and were repeatedly found in the same location [40], which we judged using relatively large sample sizes (156 ± 19 location fixes). To estimate home ranges, we used two approaches: the minimum convex polygon technique (MCP, with 100% threshold) and Kernel Density Estimates (KDEs). When performing KDEs, we used the ‘reference’ bandwidth estimation as a smoothing parameter and estimated 95% and 50% KDE [40] using the ‘adehabitatHR’ R package. Plots relating 95% KDE home range size to sampling intensity did not reach a flattening curve (see Supplementary Fig. 1). Still, the Least Square cross-validation bandwidth technique [27] showed convergence for all home ranges except for two TBFLs. Given that all home ranges had equivalent sampling and the correlation between MCP and KDE was > 0.85, we continued the analysis using only the KDE estimates.

To infer body condition, we used body mass [39] to calculate the body mass index (BMI: body mass/(wing length * 10^3^)) following Krams et al. [38]. We used linear mixed-effects models with the study sites considered a random factor to compare body conditions between the two habitat types.

## Results

### Experimental translocations

**Fig. 2:**
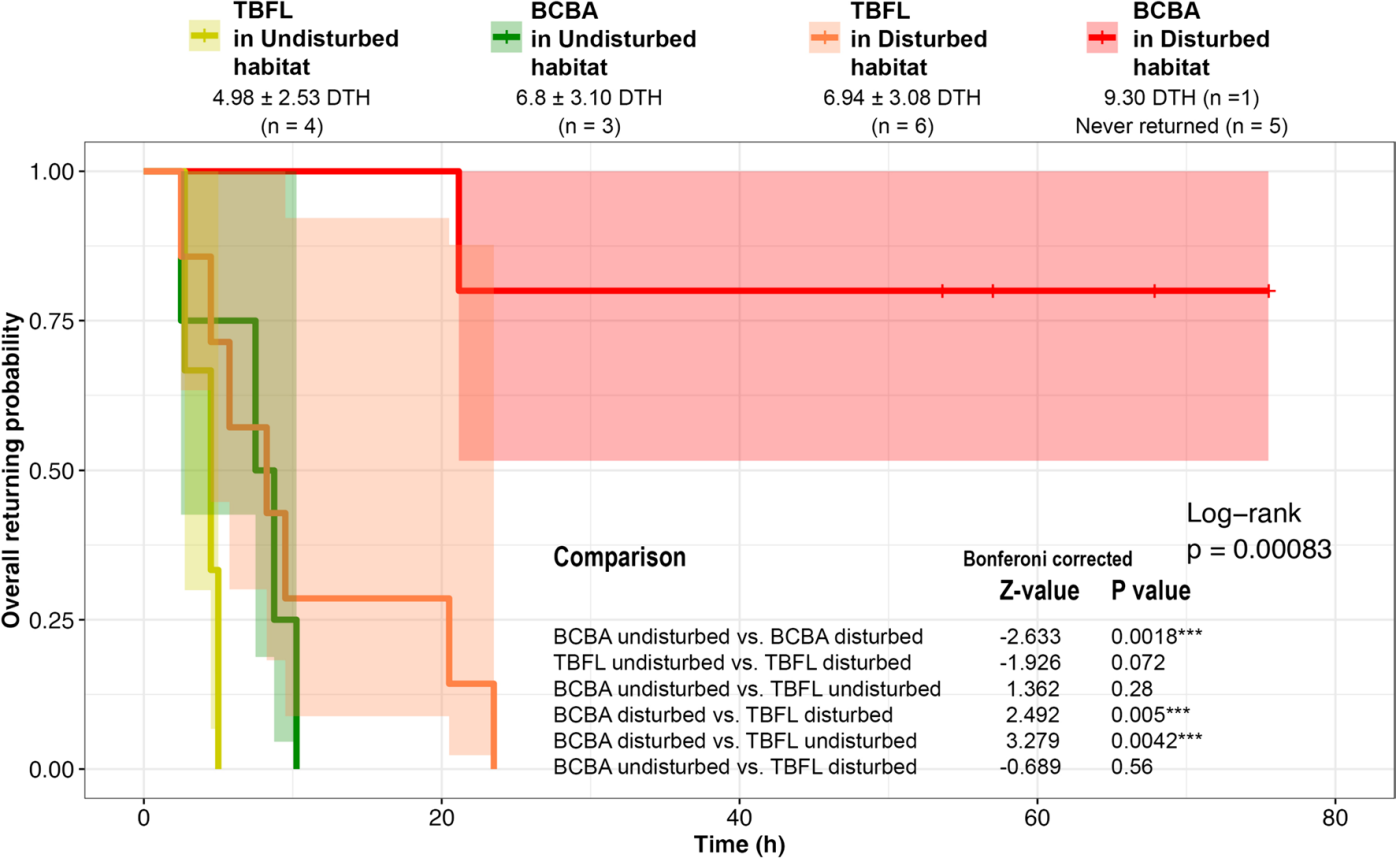
Kaplan–Meier survival curves for each bird species (Sri Lanka Brown-capped Babbler, denoted as BCBA, and Tickell’s Blue Flycatcher denoted as TBFL), showing their mean return time in different habitats), with non-returning birds right censored. *DTH* Day time hours

The two species showed contrasting results (Fig. 2), depending on the habitat they were released in. Among the BCBAs released in disturbed habitats, five out of six remained in close proximity to, or within, the release location, displaying an aversion to traversing non-forested environments. Although these five individuals occasionally wandered into open areas or rubber plantations, they quickly returned (within 15–45 min) to the location where they were initially released. After four days of observation, these individuals were recaptured and returned to their original territories. The only individual BCBA which returned to its capture location took 9.3 daytime hours. In contrast, all the three BCBAs in control experiments (released in a forest contiguous with their capture site) returned to their territories within an average of 6.8 ± 3.1 daytime hours, and a maximum of 10.5 daytime hours (see the Supplementary Fig. 2 for the trajectories of all trials).

Meanwhile, all TBFLs successfully homed within a maximum of 11.3 daytime hours, including those in control experiments (mean = 4.98 ± 2.53 daytime hours; n = 4) and disturbed habitats (mean = 6.94 ± 3.08 daytime hours; n = 6). Hence, the overall time taken to return to their capture location differed by species and habitat, with the time taken to return by BCBAs in disturbed habitats being longer than that of BCBAs in undisturbed habitats, TBFLs in undisturbed habitats, and TBFLs in disturbed habitats (see statistics in Fig. 2, and remembering the estimate of homing time for BCBAs in disturbed habitat relies on one bird). However, the difference between the return time of TBFLs in the two different habitats was not statistically significant. Interestingly, when comparing the return time across species within undisturbed habitats, the difference was not significant (see Fig. 2).

In the multivariate survival model, the interactions between bird species and the variables representing percent of rubber cover and percent of open area did not yield statistically significant results (P values > 0.05), nor was the translocation distance significant (P = 0.23). Hence, these factors were removed from the final model. The simplified model showed that the species differed in their return times, with BCBA returning more slowly than TBFL (ß = − 1.30 ± 0.58, z-value = − 2.26, P = 0.024). The percent of rubber decreased return time (ß = − 0.046 ± 0.018, z-value = − 2.59, P = 0.0095). The percent of open area had a similar negative effect on return time, although it was more variable, and thus less significant (ß = − 0.051 ± 0.029, z-value = − 1.80, P = 0.072).

### Home ranges and body condition

**Fig. 3:**
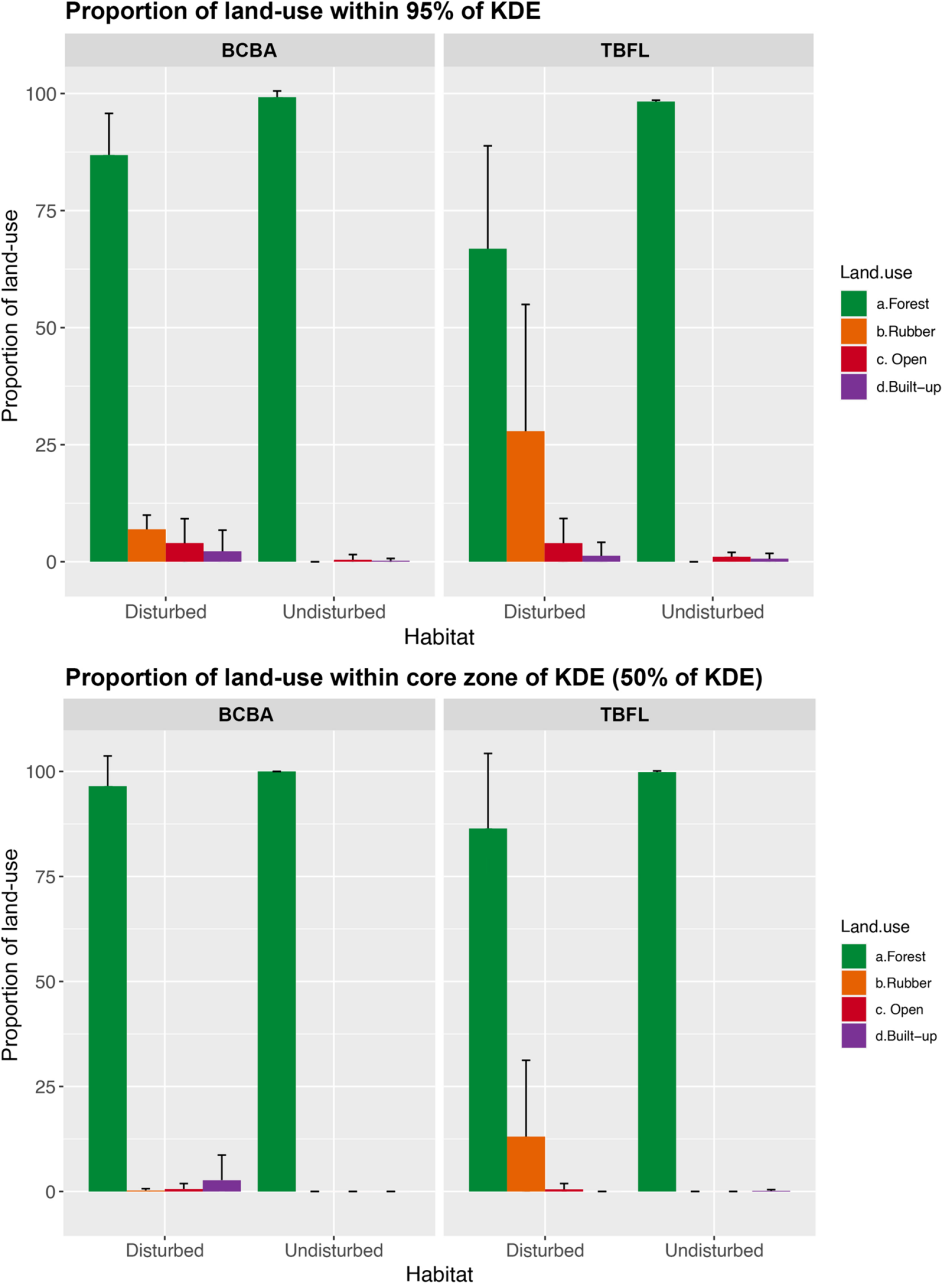
The percent cover of different land-use types inside the home-ranges of the two species in the different habitats, using the 95% KDE (Kernal Density Estimate) method of identifying home ranges (top panel) and the 50% KDE method (the “core zone”, bottom panel)

The home ranges of BCBA in disturbed areas included very low amounts of rubber (6.9 ± 3.0%, n = 5, Fig. 3). In comparison, more than 27.9 ± 27.1% (n = 8) of the TBFL’s home range in disturbed areas was rubber. When considering the core area of the home range only (50% KDE), but 0.2 ± 0.4% for BCBA and 13.1 ± 18.2% for TBFL was rubber.

**Fig. 4:**
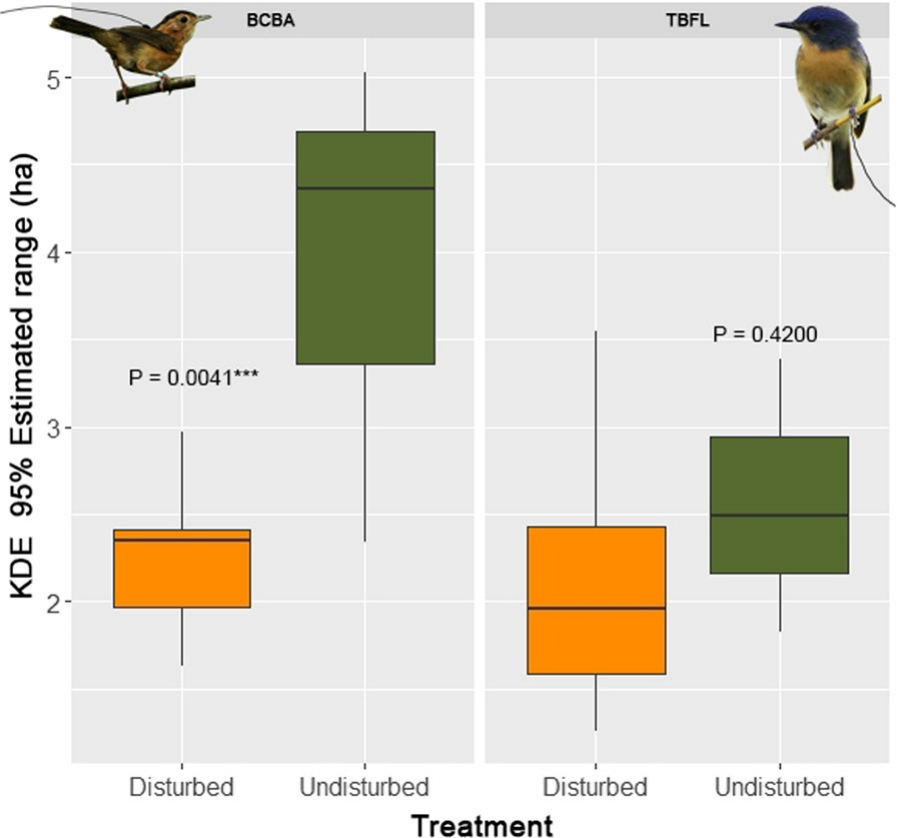
Differences for both species in home-range size between disturbed and undisturbed habitats. Home range size estimated by the 95% KDE method

There was a significant difference between home range sizes of BCBA (as measured by 95% KDE) between birds inhabiting different habitat types, with greater home range sizes within undisturbed habitats (ß = 1.735 ± 0.482, t = 3.60, P < 0.001, Fig. 4). There was no difference between habitats in the size of the home ranges of TBFL. The differences between home range sizes between habitats for the BCBA did not translate into differences in body condition. Actually, the body condition did not differ between habitats for either species (for BMI, positive coefficients and t-values indicate the measurement was higher in undisturbed habitat; BCBA: ß = 1.696^e−6^ ± 8.901^e−6^, t = 0.19, P = 0.85 and TBFL: ß = − 1.009^e−5^ ± 2.24^e−5^, t = − 0.45, P = 0.66). All the biometry data and home range data are available in the Appendix (included in Supplementary Information).

## Discussion

Overall, our results were congruent with our first hypothesis: BCBAs exhibited a marked reluctance to move through rubber plantations and open areas, whereas TBFLs encountered minimal difficulties in navigating through these habitats. As we expected for BCBA, its movements were severely hampered by the rubber matrix and open areas, with but one of six individuals translocated on rubber estates returning home. At the same time, all BCBAs in the forest returned home as fast as TBFLs. There are several potential explanations for BCBAs' strong aversion to non-forest habitats. BCBAs are understory birds with poor flying abilities. They prefer to move on the ground by hopping and staying in the understory of mature forests. They likely prefer the low light-intensity microhabitat of the forest understory and avoid higher light intensity, as has been shown for other avian understory insectivores [51]. Typically, monoculture rubber plantations have higher light intensity when compared with a forested habitat, even though rubber trees have a connecting canopy cover. Indeed, the one BCBA that successfully homed through disturbed habitats did so by moving through tea; the tea bushes may have provided the dense cover near the ground that the species prefers.

For TBFL, homing success did not differ significantly between undisturbed and disturbed habitats (Fig. 2). This may be due to either the species' high flight performance—TBFLs have been observed to cover distances of 80 m in a single flight (personal observation)—or the generalist nature of the species' habitat preferences. And yet, even though rubber and open matrices were relatively permeable to TBFL movements, they did not move deep into rubber, but rather preferred to move along the border of rubber and disturbed forest (i.e., the last line of rubber trees before the edge). Indeed, the result that an increasing cover of rubber decreases the return time of birds was not specific to species, applying both to TBFL and BCBA (i.e., there was no significant interaction in the model). It would be interesting to see in future studies whether retaining some pockets of natural trees in rubber landscapes would provide “stepping stones” for TBFLs, as shown in pasture landscapes for other species [8, 19]. Regardless, our study shows that even for forest generalist species, detailed data on movement ecology is critical for the formulation of effective conservation management plans.

Our analysis of home range data reveals that BCBAs in disturbed lands were largely restricted to small fragments and thus had much smaller foraging areas than within protected areas. The effect size was large, with home ranges in protected areas being, on average, nearly twice as large as those in disturbed regions (Fig. 4). There were no differences in home range size between habitats for TBFLs (although sample size in undisturbed forest was low at only three birds). Compared to forested habitats, we observed TBFLs performing foraging events within the rubber and open area matrices, especially at dusk (after 18:00), and the high visibility of insects in these areas at these times could be a reason for this [2]. TBFLs were similar to BCBAs in not including large non-forest areas in their home ranges. Regarding the estimated home ranges of both species in disturbed habitats, 75% consisted of forested habitats, and rubber represented only 7% of BCBA and 28% of TBFL home ranges. When considering the core zone of the home ranges, the proportion of non-forested areas was further reduced to 0.2% in BCBA and 13% in TBFL. Thus, information on the natural habitat selection of these two bird species was complementary to that gained through the translocations, clearly showing the adverse effects of rubber. This indicates that translocation experiments can serve as a valuable tool in providing data for evidence-based conservation management.

We were surprised that BCBAs did not have significantly poorer body conditions in disturbed areas, given their substantially smaller home ranges and hence presumably lesser food resources. This may be due to unmeasured variables like the actual age of the individuals or food availability at their hatching territories—perhaps some individuals had lived elsewhere before and had only recently moved to these sites, so that the poor conditions had not yet affected them. Alternatively, disturbed secondary patches of forest surrounded by rubber may actually have high food availability [54, 56]. We hope future studies will further investigate how home range size and food availability affects bird fitness in these areas.

Several limitations warrant discussion to assess their impact on the results of our study. Primarily, the collection of home range data occurred during the COVID-19 pandemic, compromising the standardisation of sampling procedures. Specifically, some home range assessments were conducted during singular site visits, while others were performed across multiple visits. In addition, a minority of the home range measurements were conducted subsequent to translocation events. However, tests comparing singular to multiple visits, or individuals that were translocated to those that were not, showed no evidence that these factors influenced our results (see the “home range surveys” section of the methods). We also conducted home range measurements over multiple seasons. Territory size may vary seasonally [17, 32], but we believe seasonality should not be a large confounding variable for this study, as most home-range measurements (17 of 23) included sampling during both the non-breeding and the breeding season (in the breeding season, we only took data if the observed individuals did not exhibit signs of breeding themselves). Further, all home range measurements had a reasonable number of location fixes to generate home range estimates [53].

The translocation experiments also could be influenced by some confounding variables that require inspection. Betts et al. [6] in their review on animal translocations discuss six potential confounding variables: capture site quality, physiological condition of the subjects, release site quality, quality of the intervening habitat between capture and release site, release site familiarity, and extreme stress elicited by the experiment. To mitigate potential biases introduced by these confounding variables, we implemented several precautionary measures. To account for variability in capture site quality, all individual birds for the disturbed habitat treatments were captured in secondary forest patches that were approximately uniform in terms of vegetation composition and level of disturbance. The birds in forests were indeed captured in more mature forests; despite this, we consider it unlikely that the very distinctive results observed in the different habitats can be attributed to these birds exerting greater efforts to return to their high-quality territories. Birds in disturbed habitats were evidently attempting to return, as evidenced by their numerous brief forays into the surrounding matrix before retreating back to their initial release locations. To control for physiological differences between individuals, both body weight and stress conditions were measured, with no significant differences found even between the different habitats. Release locations were standardized to be very small patches from which the birds would want to move away. We also checked the intervening matrix for conspecific individuals, as the calls and songs of conspecific members can interfere with their behavior [66, 67], however, only one TBFL was observed in one trial.

While most potential biases were controlled for by standardization, as mentioned above, others were more difficult to avoid. Betts et al. [6] argued that if some individuals are more familiar with their release site than others, this could affect results (e.g., they might be able to home more rapidly; [12, 70]). Influenced by ethical concerns to reduce impacts on birds, all of our translocation distances were relatively short (< 500 m), below the average distances of past studies [15], although much above the average width of a home range of the species with the highest flight capabilities, TBFL (207.7 ± 43.7 m). We think it is unlikely that birds had experience with their release locations, mainly since these locations were often across matrices that the birds were found to avoid. Regarding the potential stress of translocations, the control trials showed that birds that undergo translocations are not negatively affected by the translocation process and are able to return home through contiguous forests. Also interested in understanding translocation-related stress, Volpe et al. [63] did a study in which they compared the movement and habitat selection of translocated birds to those on their territories. They found that translocated birds moved faster than usual but that both groups of birds showed avoidance of non-forested habitats. This is reminiscent of our results, in which the avoidance of non-forest habitat, and rubber plantations in particular, seen in the home-range study was similar to that observed in the translocations.

## Conclusions and conservation implications

To conclude, the forest specialist species in this study could not return when translocated across either rubber or open landscapes, and even the forest generalist avoided going deep into rubber. Similar results have been found in earlier studies where roads or conifer plantations act as consistent barriers to the movement of forest specialist birds [62].

Regarding BCBA in particular, the species has been identified as potentially sensitive to human threats, given that its abundance and elevational range size are below average for endemics in Sri Lanka [57]. In terms of its survival in rubber plantations, we suggest incentivizing “jungle rubber”, which allows some natural understory regrowth [7]. Another strategy would be intercropping with crops with different vegetation structures, such as tea, coffee and cacao trees, to create a structurally complex and shady understory [23, 30]. Improving landscape connectivity through corridors through rubber [5, 64] that include understory plant species could also facilitate the movements of this species and other understory habitat specialists [18]. Further, enriching these corridors to mimic the shady microclimatic conditions present in structurally complex mature forest will benefit many understory species, as has been shown by [49], producing thermal refugia for species that are sensitive to climate warming [36].

Overall, although based on a modest sample size, our study emphasizes that while agroforests represent a way of sequestering carbon [1], they may not be favourable for biodiversity if they remain monocultures [16, 29]. New creative solutions are required to conserve forest specialist wildlife in these landscapes.

### Supplementary Information

Supplementary Material 1: Appendix 1, a database with raw data from all observed individuals.

Supplementary Material 2: Document including Supplemental Table 1 (sampling details for home range study), Supplemental Figure 1 (home range convergence plots), and Supplemental Figure 2 (movement trajectories of all translocated individuals).

## Data Availability

The trajectories of all translocated individuals are available in Supplementary Figure 2, and the data from all observed individuals in included in Appendix 1, in the Supplementary Information.

## Abbreviations

BCBA: Brown-capped Babbler
TBFL: Tickle’s Blue Flycatcher
KDE: Kernel Density Estimates
MCP: Minimum Convex Polygon
BMI: Body Mass Index
HWI: Hand-Wing Index
ERC IOBSL: Ethical Research Committee Institute of Biology Sri Lanka
