# Supplementary material for: Rubber plantations are impermeable to an avian understory specialist in Sri Lanka
Source: Mov Ecol. 2024 Jun 17;12:47. doi: 10.1186/s40462-024-00484-8 (PMC11181654; doi:10.1186/s40462-024-00484-8)
Supplement: Supplementary file 2 — Supplementary Material 2: Document including Supplemental Table 1 (sampling details for home range study), Supplemental Figure 1 (home range convergence plots), and Supplemental Figure 2 (movement trajectories of all translocated individuals). [file 40462_2024_484_MOESM2_ESM.docx]

**Rubber plantations are impermeable to an avian understory specialist in Sri Lanka.**

**SUPLEMENTAL MATERIAL**

Salindra K. Dayananda, Harsha F. Athukorala, Indika Peabotuwage, Chandralal Kumara, Tharindu Ranasinghe, Dhammithra Samarasinghe, Ana Gouveia, Sarath W. Kotagama, Christos Mammides, Aiwu Jiang, Eben Goodale

| **Table of contents** | | **Page** |
| --- | --- | --- |
|  | **Supplemental Table 1.** Home range survey sampling, broken down by season, through the years of 2019-2022…………………………………………………………………………… | 2 |
|  | **Supplemental Figure 1.** Home range analysis convergence, showing smoothing plot and asymptotes for each individual of both species, using least-square cross validation (LSCV) techniques and generated with the ‘adehabitatHR’ package in R (B=BCBA and T=TBFL)…………………………………………………………….. | 4 |
|  | **Supplemental Figure 2**. Movement trajectories of all the individuals subjected to translocations (Dark green = Secondary Forest, Light Green = rubber, pink = Tea/ Open, brown = Other land-use types)………………………………………………... | 9 |

**Supplemental Table 1.** Home range survey sampling, broken down by season, through the years of 2019-2022.

| **ID** | **Jan** | **Feb** | **Mar** | **Apr** | **May** | **Jun** | **Jul** | **Aug** | **Sep** | **Oct** | **Nov** | **Dec** | **Days in breeding season** | **Days in non-breeding season** | **Total**  **days** |
| --- | --- | --- | --- | --- | --- | --- | --- | --- | --- | --- | --- | --- | --- | --- | --- |
| **B8** | 1D | ½D | ½D |  |  |  |  |  |  |  | 1D | 1D | 2 | 2 | 4 |
| **B9** |  |  | 1D |  |  |  |  |  |  |  |  | 2D* | 1 | 2 | 3 |
| **B10** |  |  | ½D |  |  |  |  | 1D | 1D | ½D |  |  | 2 | 1 | 3 |
| **B12** |  |  |  | 3D* |  |  |  | 1D |  |  |  |  | 3 | 1 | 4 |
| **B13** |  |  |  | 2 & ½D |  |  |  | 1D |  |  |  |  | 2.5 | 1 | 3.5 |
| **B14** | ½D |  |  | 1D |  |  |  | ½D |  | 1D |  |  | 2.5 | 0.5 | 3 |
| **B15** | 1D |  |  |  |  |  |  | 2D* |  |  |  |  | 1 | 2 | 3 |
| **B17** | 2 & ½D |  |  |  |  |  |  |  |  |  | 1D |  | 2.5 | 1 | 3.5 |
| Total sampling period for the BCBA individuals in undisturbed habitats | | | | | | | | | | | | | **16.5** | **10.5** | **27** |
| B5 | 1D |  |  |  |  |  |  |  |  | ½D | 1 & ½D |  | 1.5 | 1.5 | 3 |
| B11 |  | 3D* | 1D |  |  |  |  |  |  |  |  |  | 4 | 0 | 4 |
| B19 |  |  | 3D* |  |  |  |  |  |  |  |  |  | 3 | 0 | 3 |
| B21 |  |  |  |  |  |  | 2D | 2D |  |  |  |  | 0 | 4 | 4 |
| B22 |  |  |  |  |  |  | 2D* | 2D |  |  |  |  | 0 | 4 | 4 |
| Total sampling period for the BCBA individuals in disturbed habitats | | | | | | | | | | | | | **8.5** | **9.5** | **18** |
| **Total** | | | | | | | | | | | | | **25** | **20** | **45** |
| **T8** |  | 2D* |  |  |  |  |  |  |  |  | 1D |  | 2 | 1 | 3 |
| **T10** |  | ½D |  |  |  |  |  |  |  |  | ½D | 2D | 0.5 | 2.5 | 3 |
| **T11** |  | 3D* |  |  |  |  |  |  |  |  |  |  | 3 | 0 | 3 |
| Total sampling period for the TBFL individuals in undisturbed habitats | | | | | | | | | | | | | **5.5** | **3.5** | **9** |
| T2 |  |  |  |  |  |  |  |  |  |  | 3 & ½D* |  | 0 | 3.5 | 3.5 |
| T3 | 3D* |  |  |  |  |  |  |  |  |  |  |  | 0 | 3 | 3 |
| T5 |  | 2D* |  |  |  |  | 1D |  |  |  |  |  | 3 | 0 | 3 |
| T7 |  | ½D |  |  |  |  | 2D* | ½D |  |  |  |  | 3 | 0 | 3 |
| T12 |  | 1 & ½D |  |  |  |  |  |  | 1 & ½D |  |  |  | 1.5 | 1.5 | 3 |
| T16 |  |  |  |  |  |  | 1D | 1 & ½D |  | ½D |  |  | 2.5 | 0.5 | 3 |
| T20 |  |  |  |  |  |  | ½ & 1½D* | 1D |  |  |  |  | 3 | 0 | 3 |
| Total sampling period for the TBFL individuals in disturbed habitats | | | | | | | | | | | | | **13** | **8.5** | **21.5** |
| **Total** | | | | | | | | | | | | | **18.5** | **12** | **30.5** |

Notes: Sampling effort denoted by D = Days (1/2D = 6 hours sampling, * denote number of consecutive days), light colour denote full breeding range and dark colour denote peak breeding months for babbler (yellow) and flycatcher (blue). None of the observed individuals during the breeding period showed any signs of active nesting. Peak breeding for BCBA is from Mar-April and for TBFL peak breeding is from March to June (Kotagama & Ratnavira, 2017, [Billerman et al. 2022](#_ENREF_7)). Bolded column IDs represent individuals sampled at undisturbed habitats. Grey shaded column IDs represent the individuals that were subjected to translocations.

**Supplemental Figure 1.** Home range analysis convergence, showing smoothing plot and asymptotes for each individual of both species, using least-square cross validation (LSCV) techniques and generated with the ‘adehabitatHR’ package in R (B=BCBA and T=TBFL).


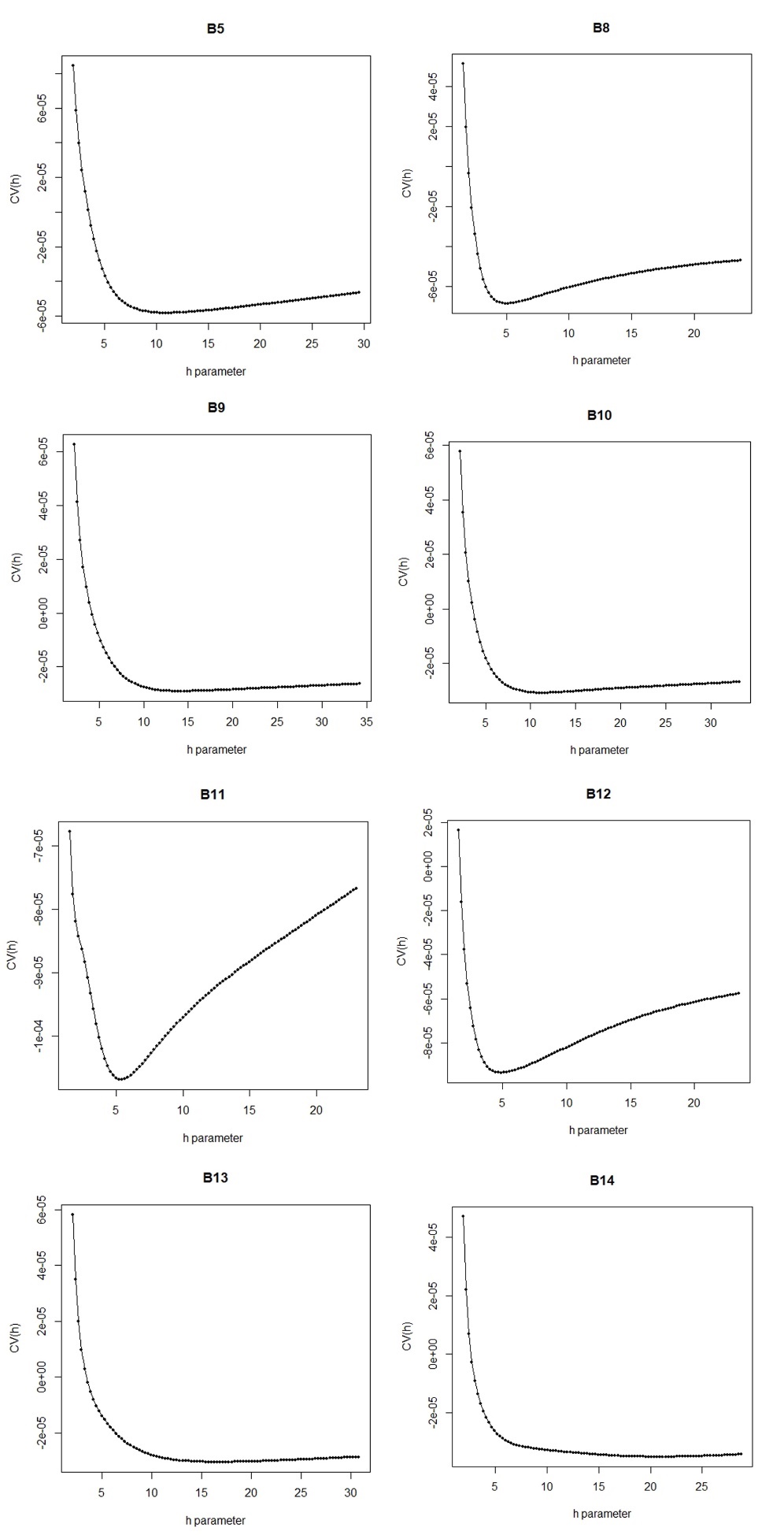


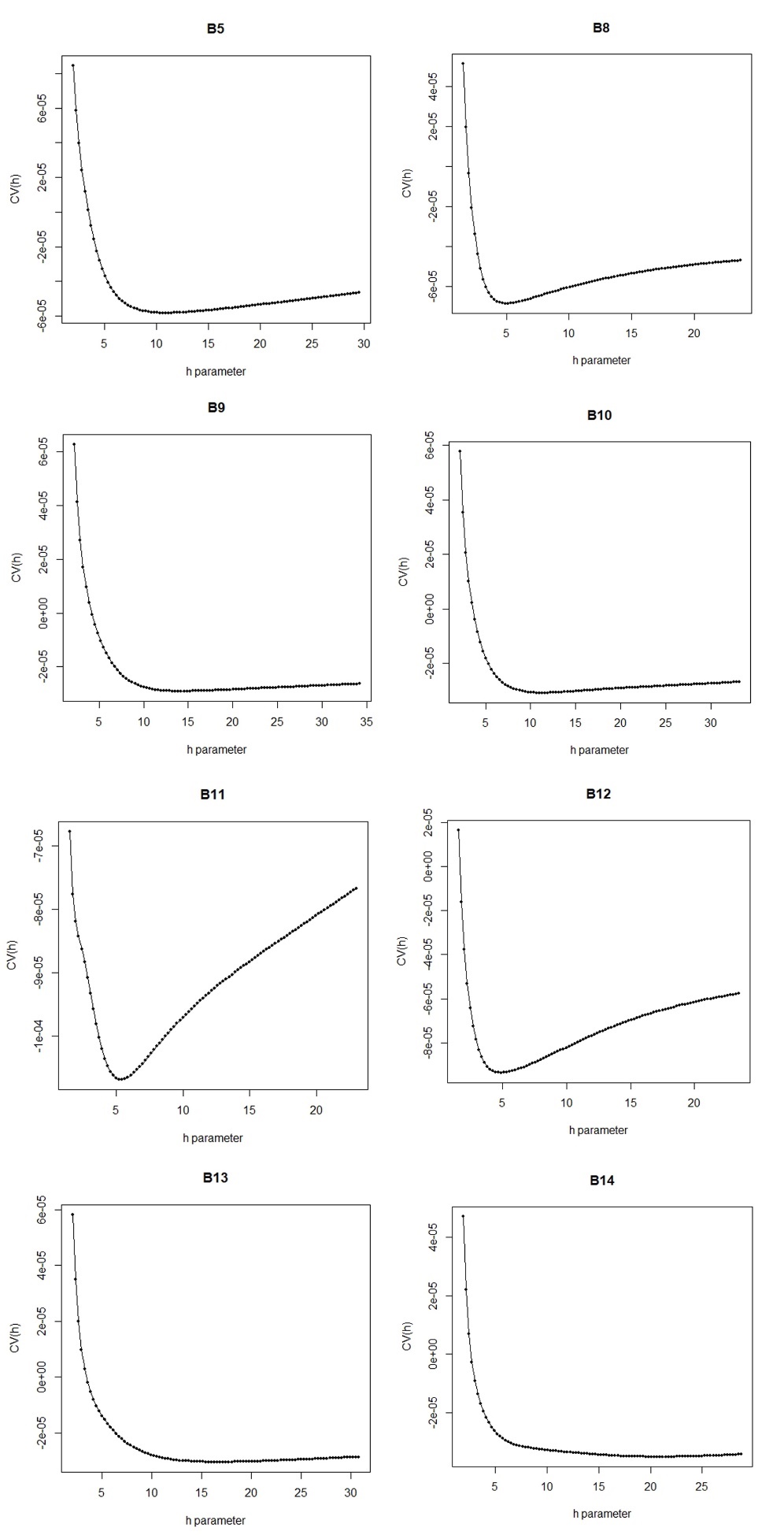


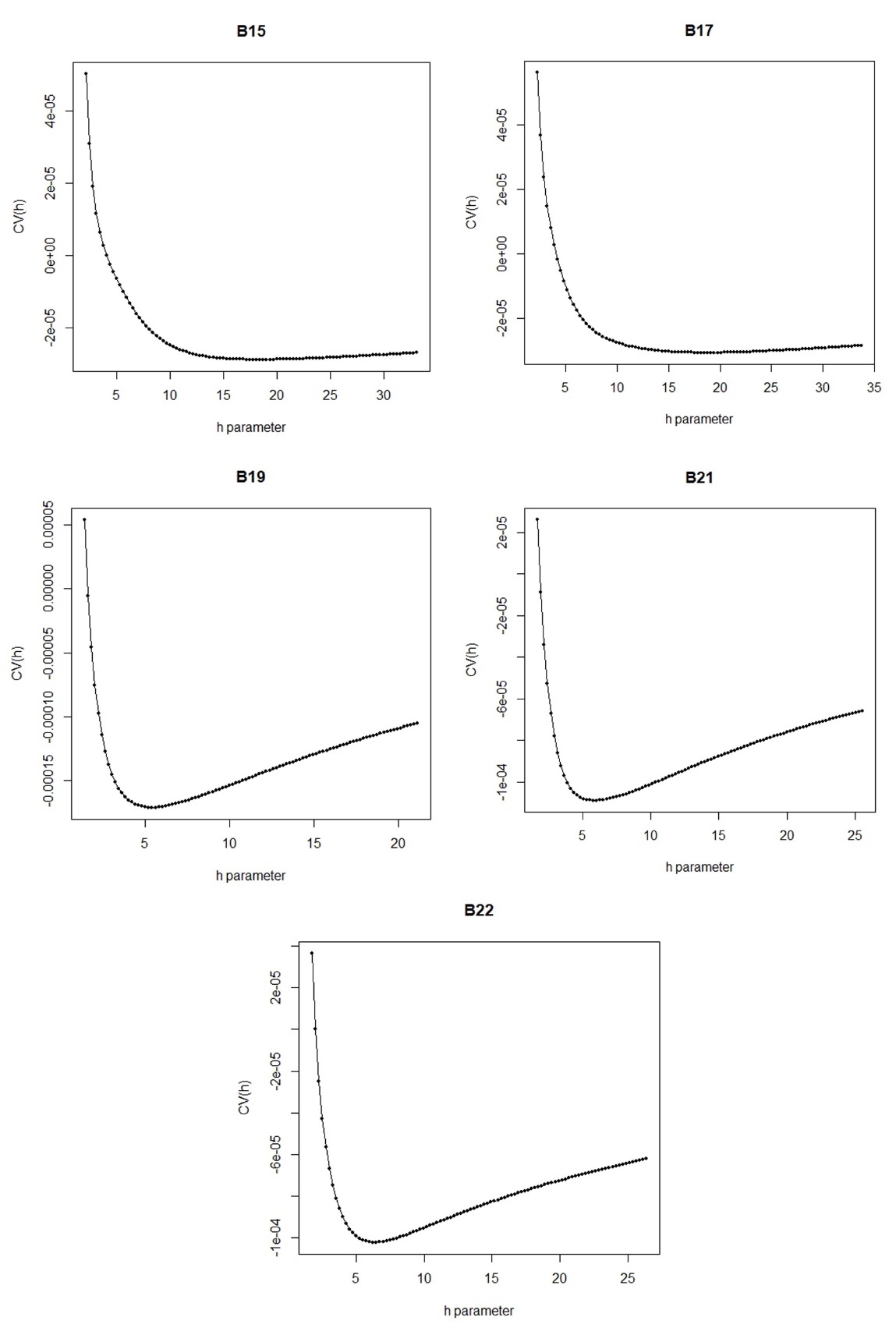


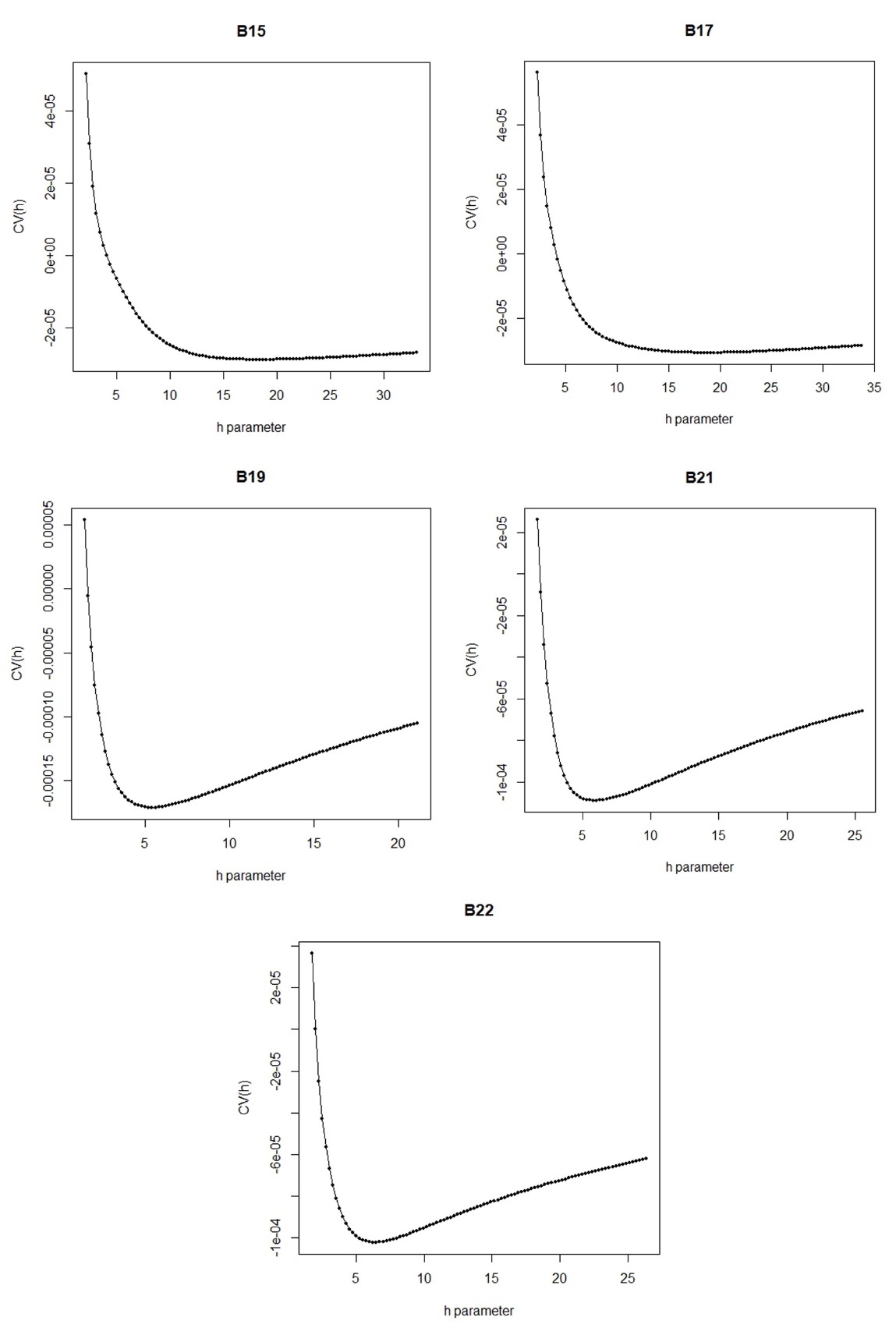


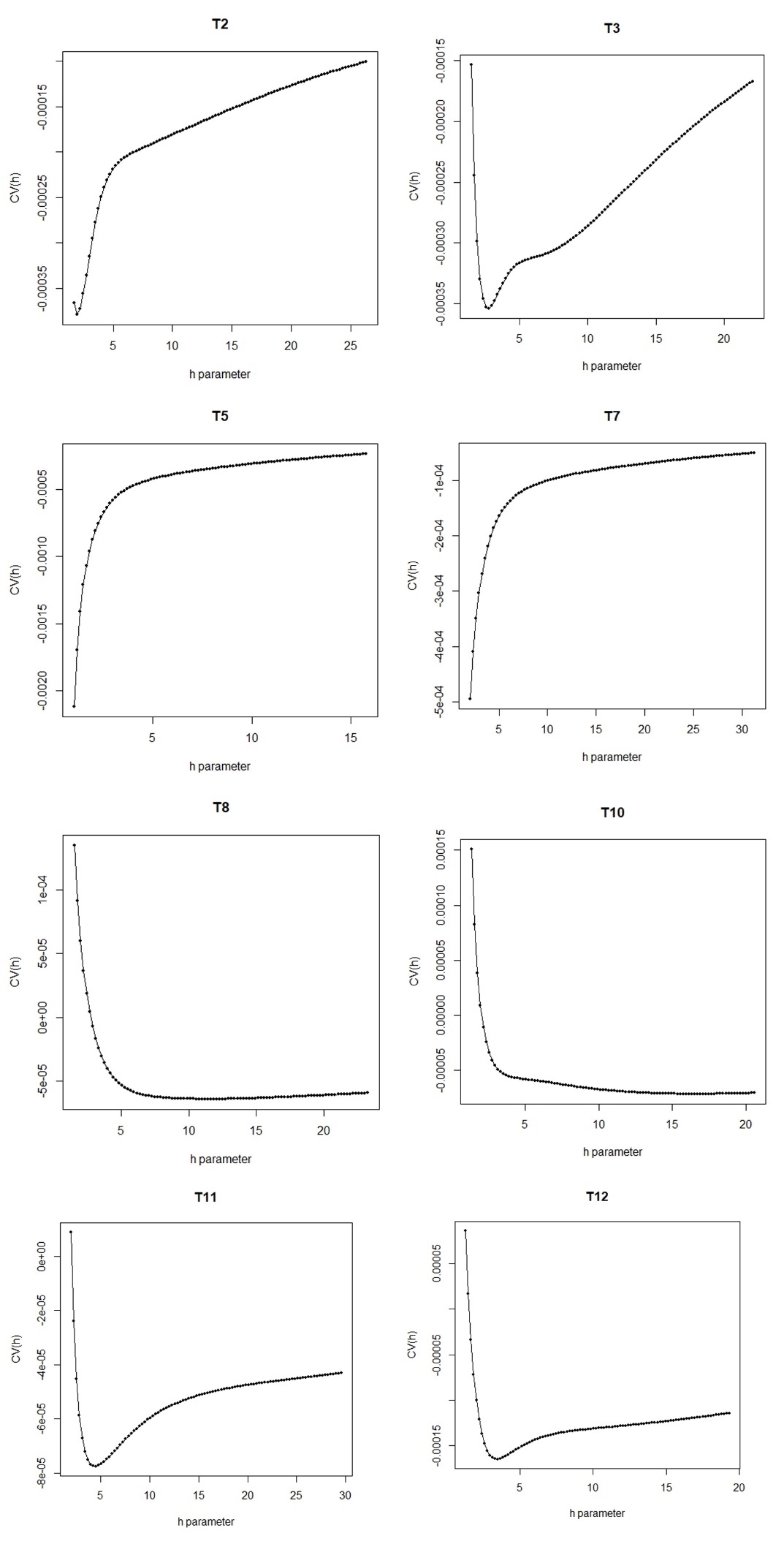


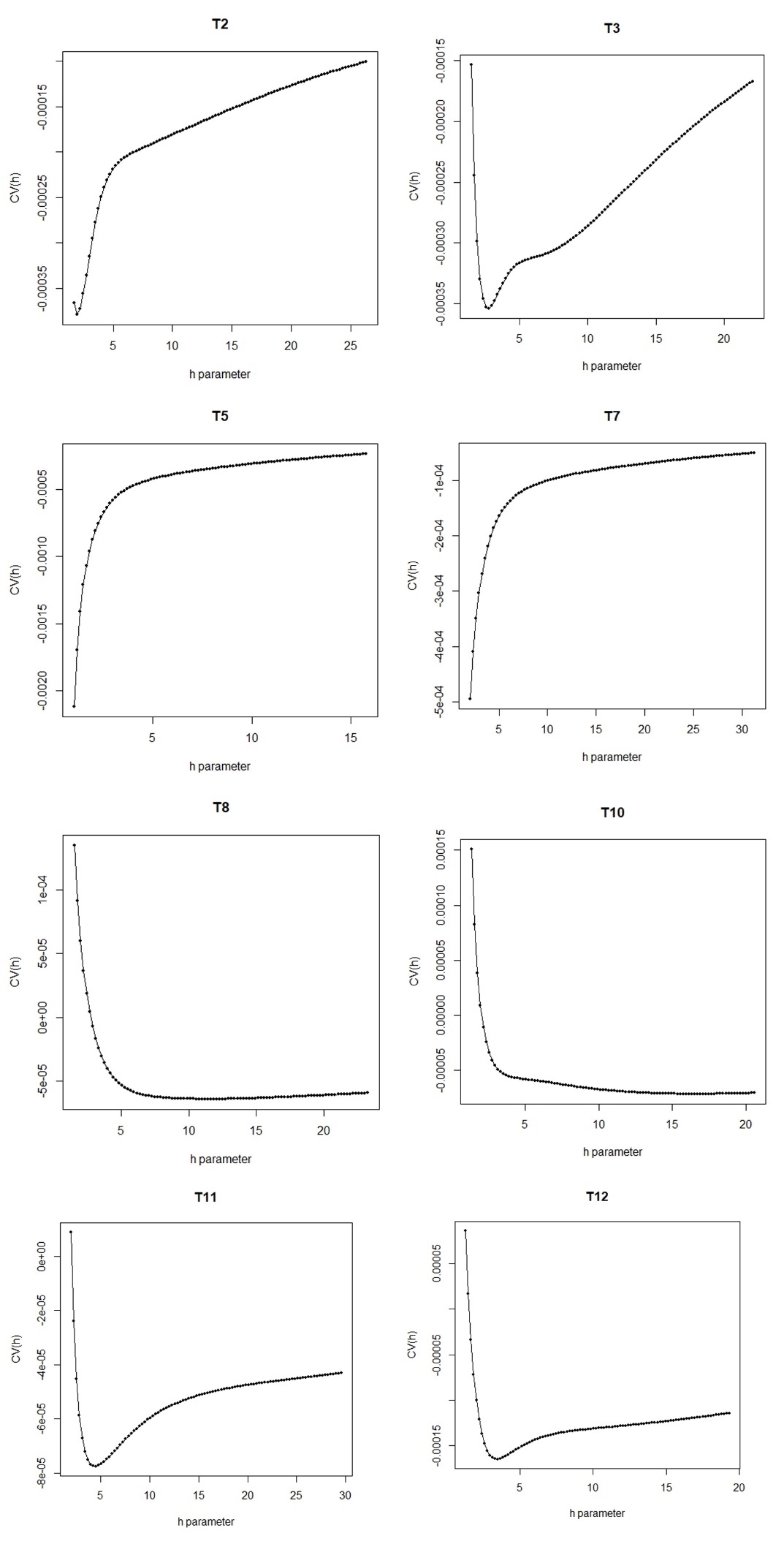


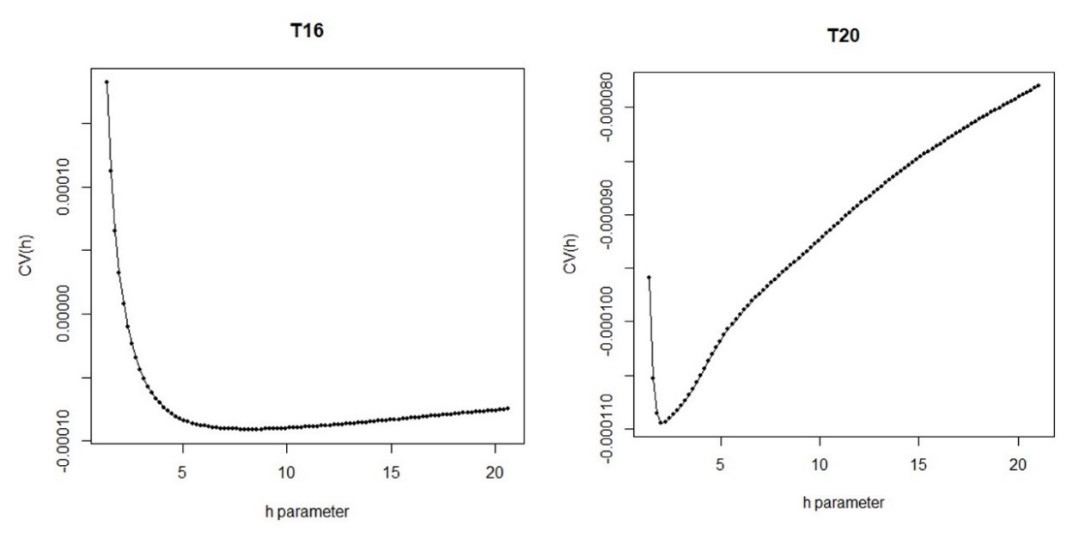


**Supplemental Figure 2**. Movement trajectories of all the individuals subjected to translocations (Dark green = Secondary Forest, Light Green = rubber, pink = Tea/ Open, brown = Other land-use types).


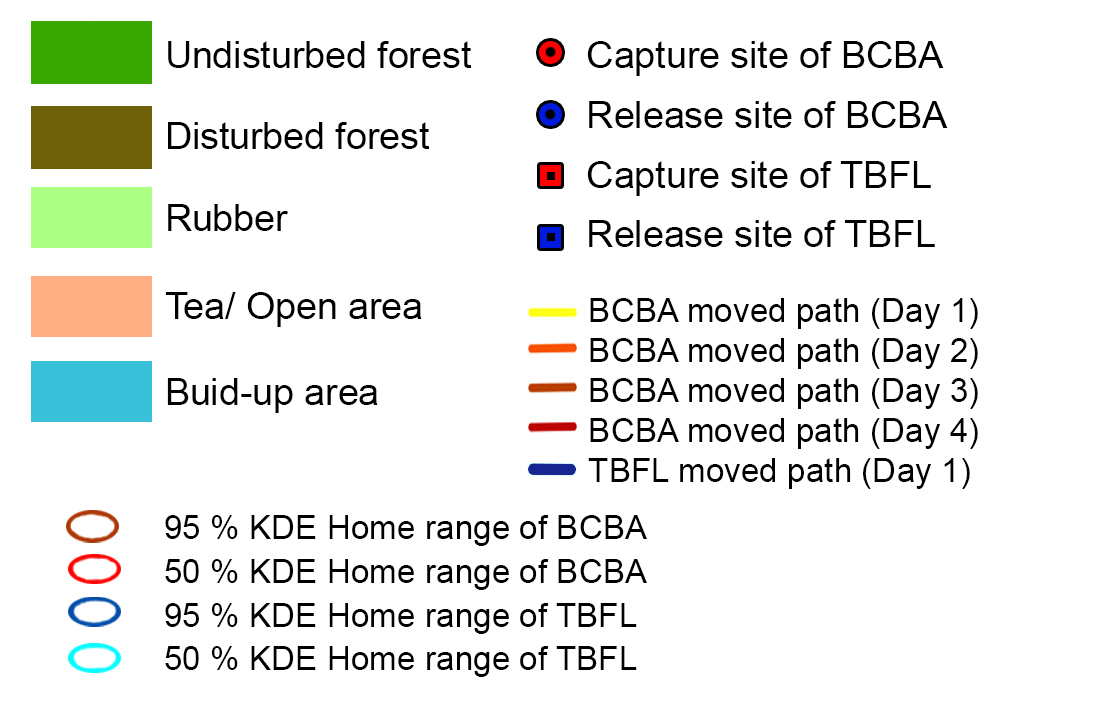


Common legend for all the map figures in the supplemental figures. For information on the individual babblers (B) or flycatchers (T), please see Appendix 1.

**Movement trajectories of translocated Sri Lanka Brown-capped Babblers in both undisturbed and disturbed habitats**


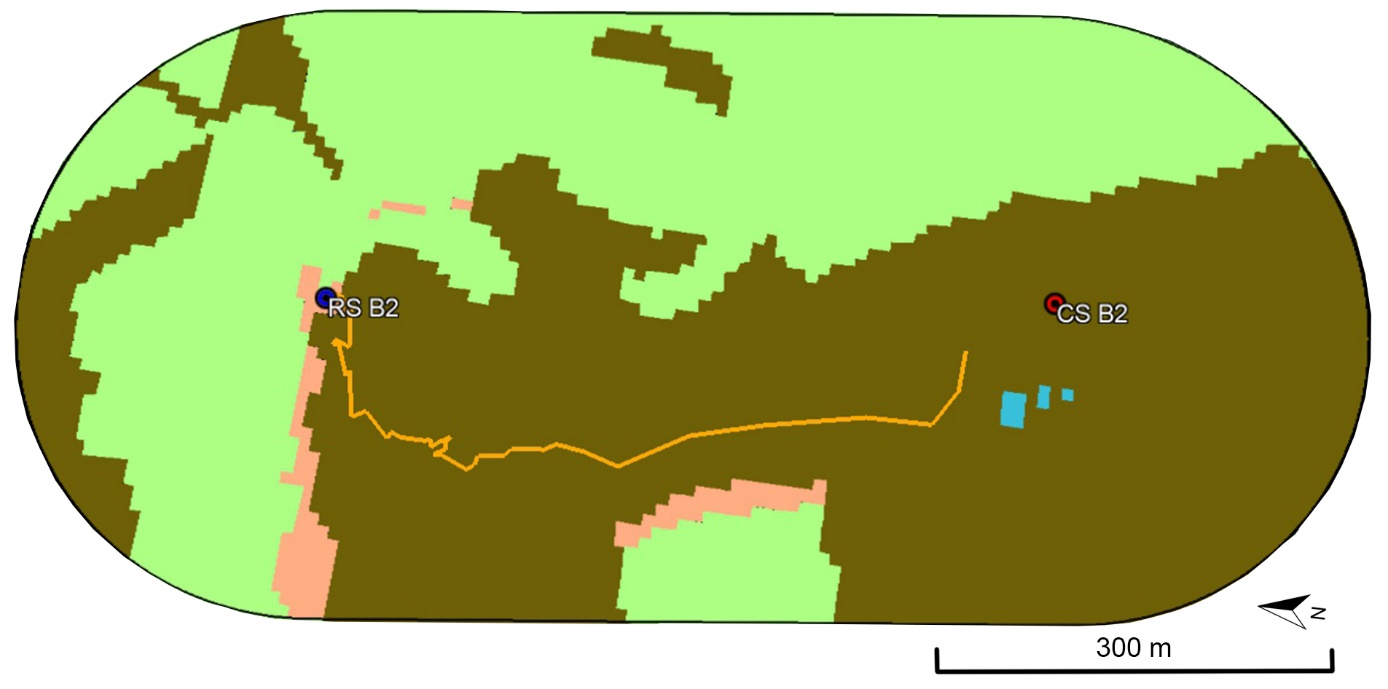


Figure S 2.1: Moved path of B2 individual in Veeoya


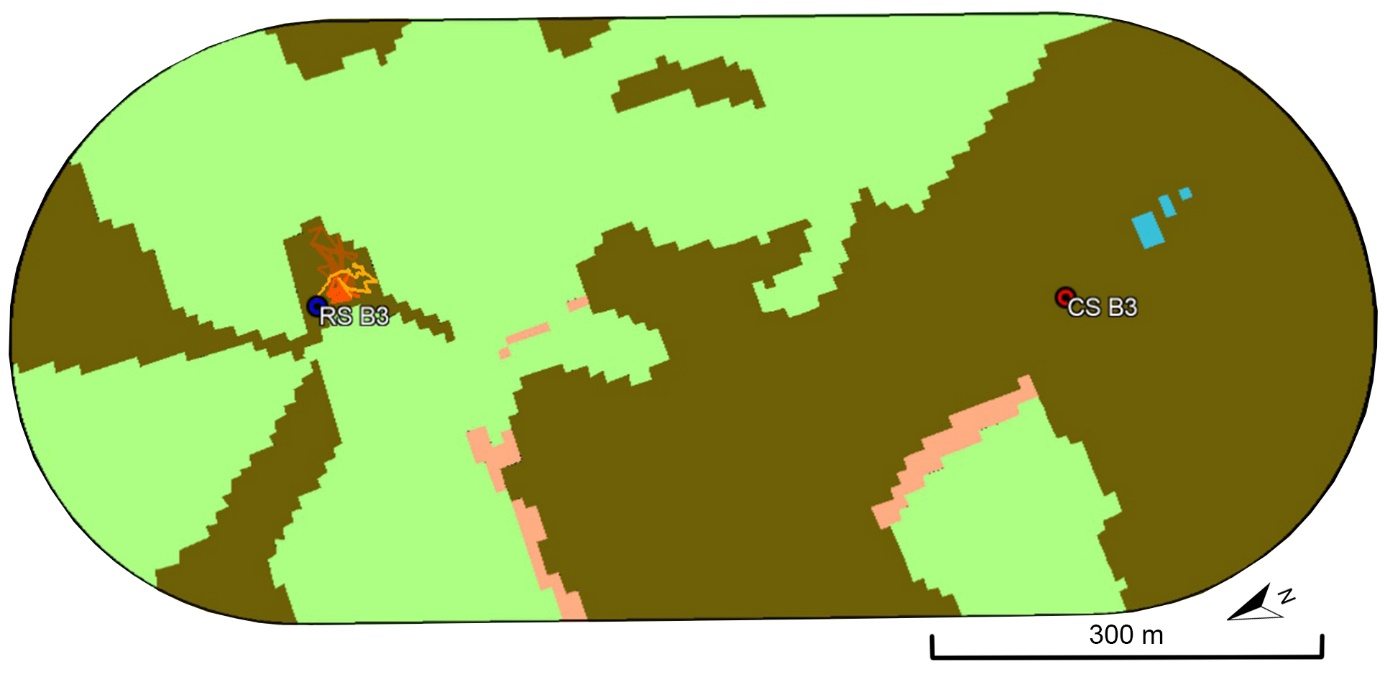


Figure S 2.2: Moved path of B3 individual in Veeoya


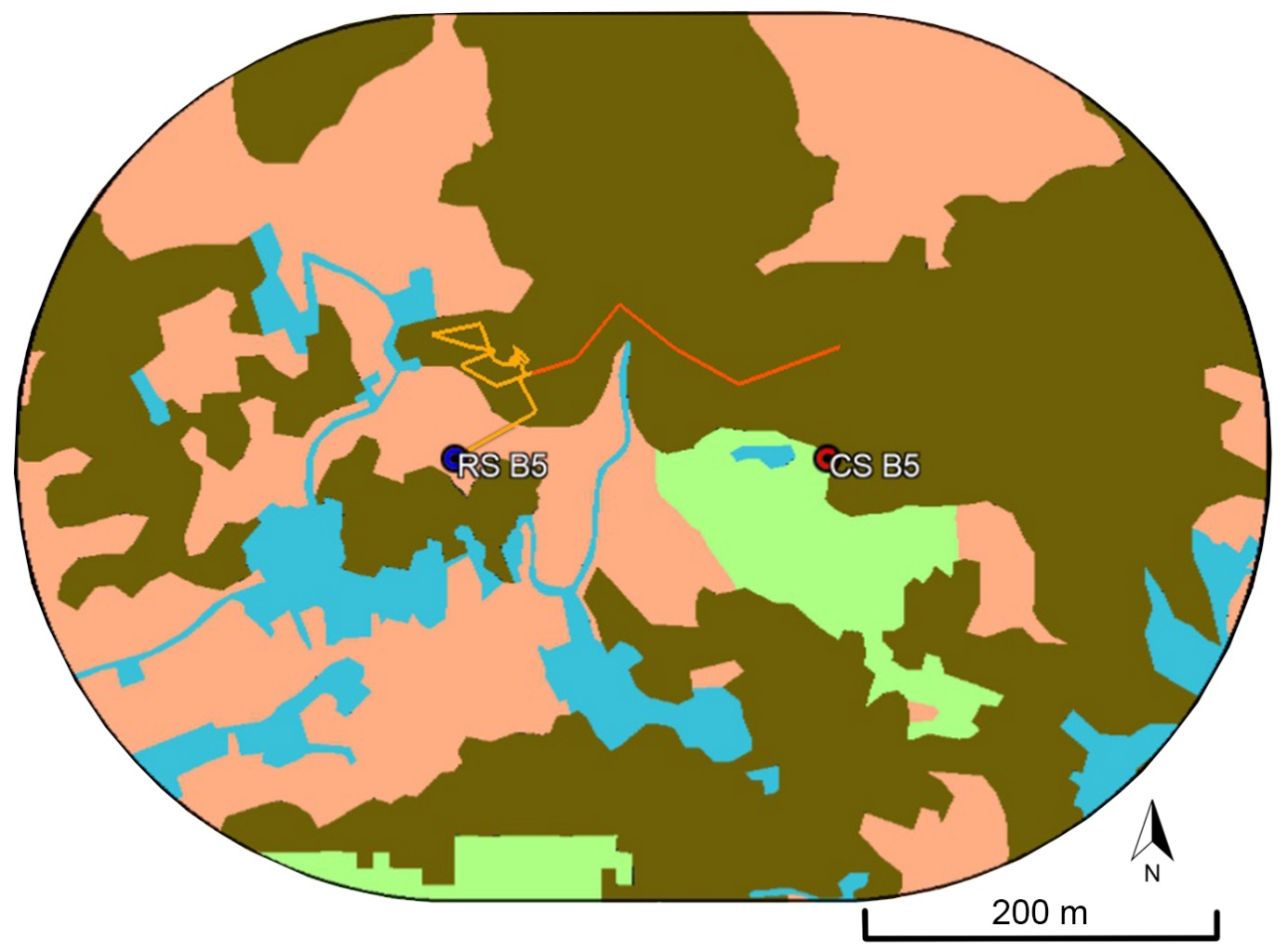


Figure S 2.3: Moved path of B5 individual in Halgolla


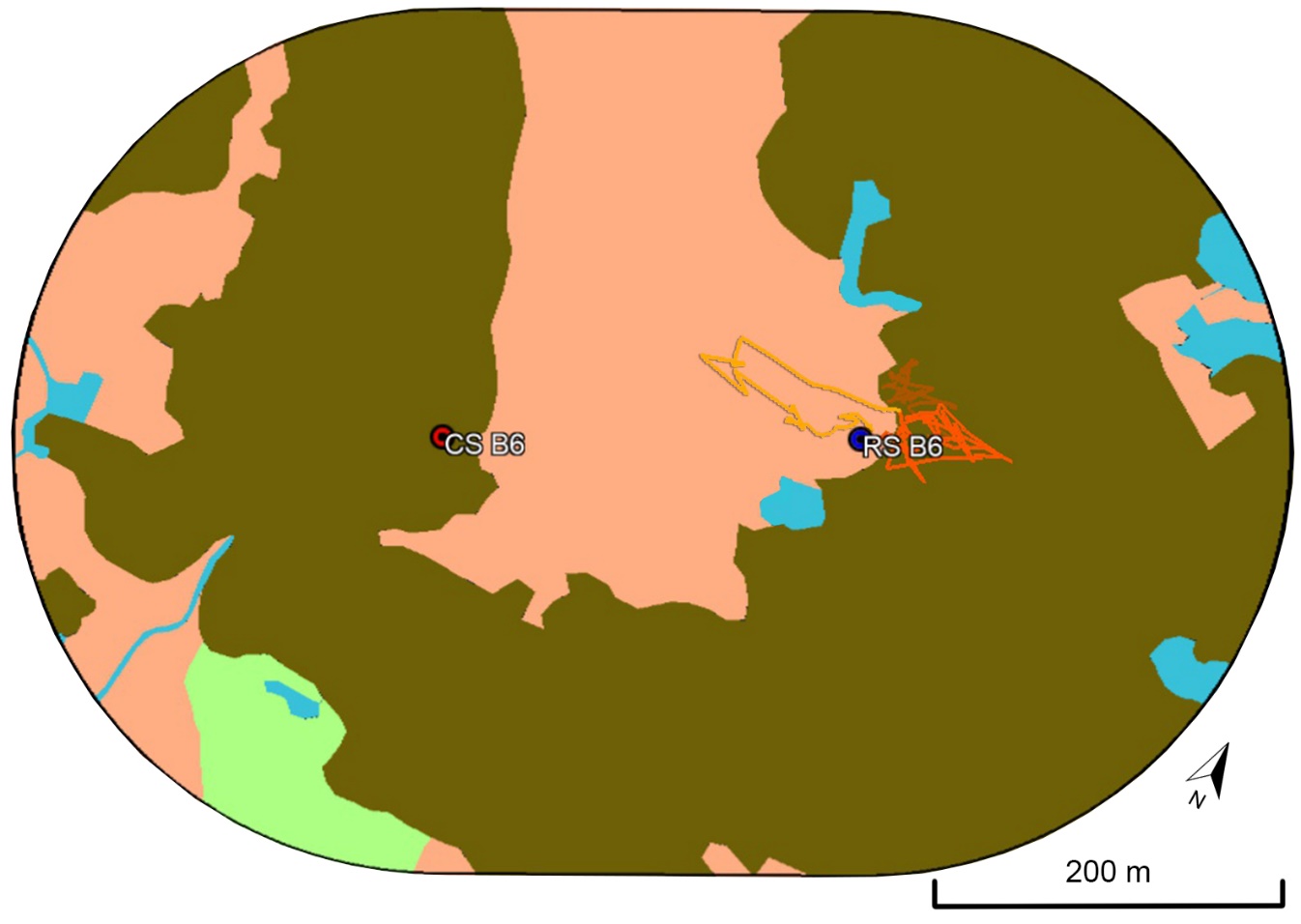


Figure S 2.4: Moved path of B6 individual in Halgolla


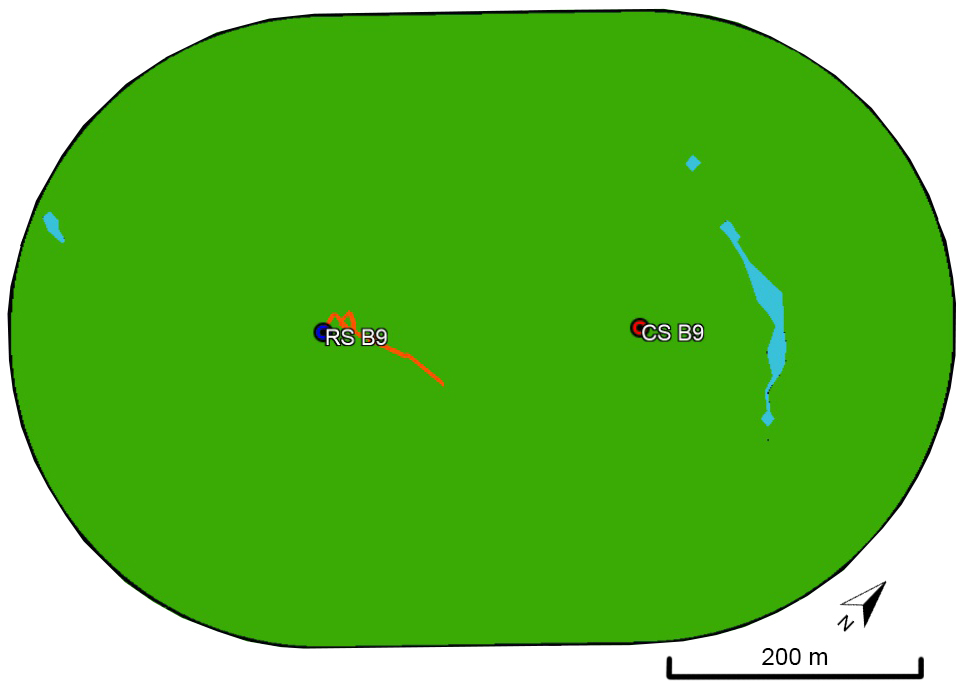


Figure S 2.5: Moved path of B9 individual in Kithulgala Forest Reserve


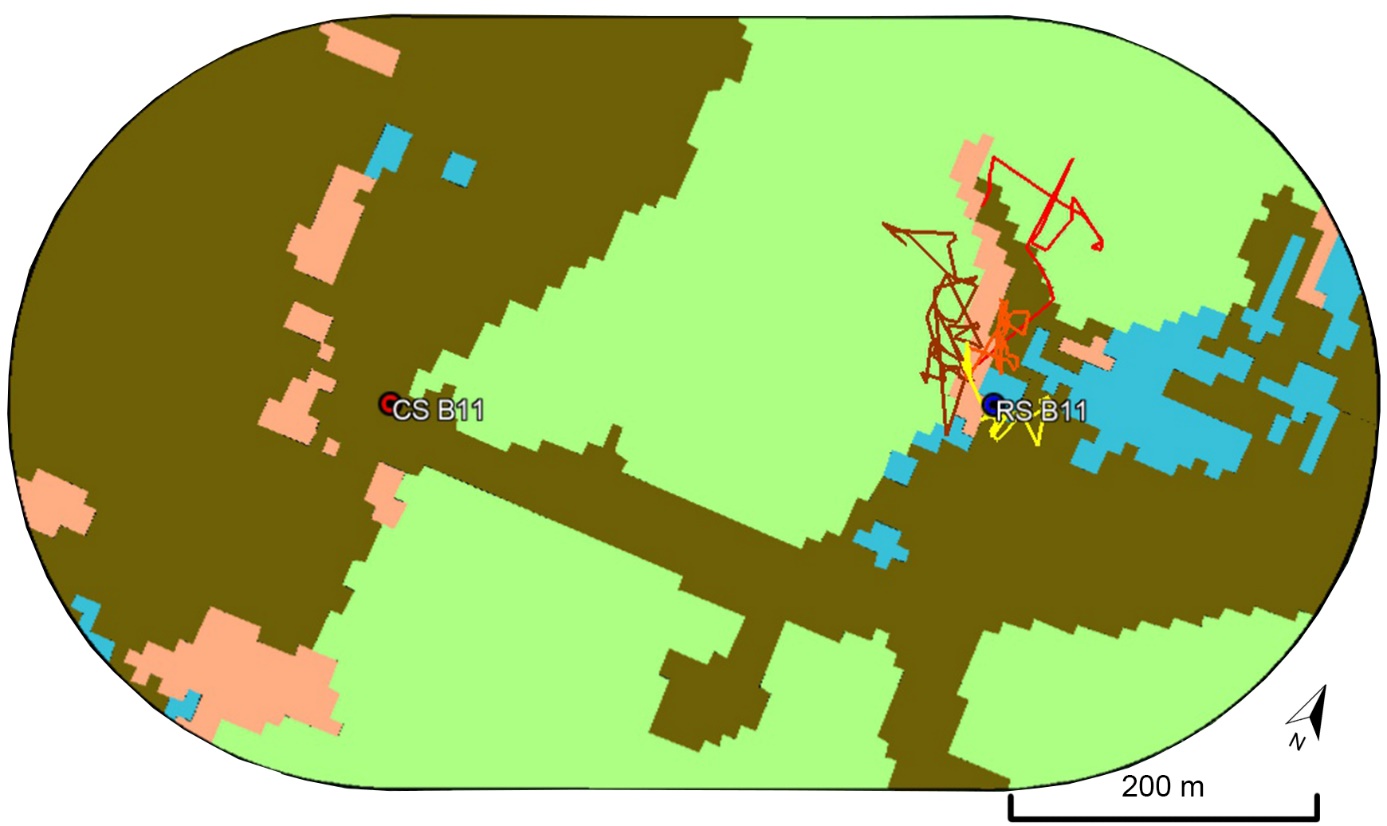


Figure S 2.6: Moved path of B11 individual in Veeoya


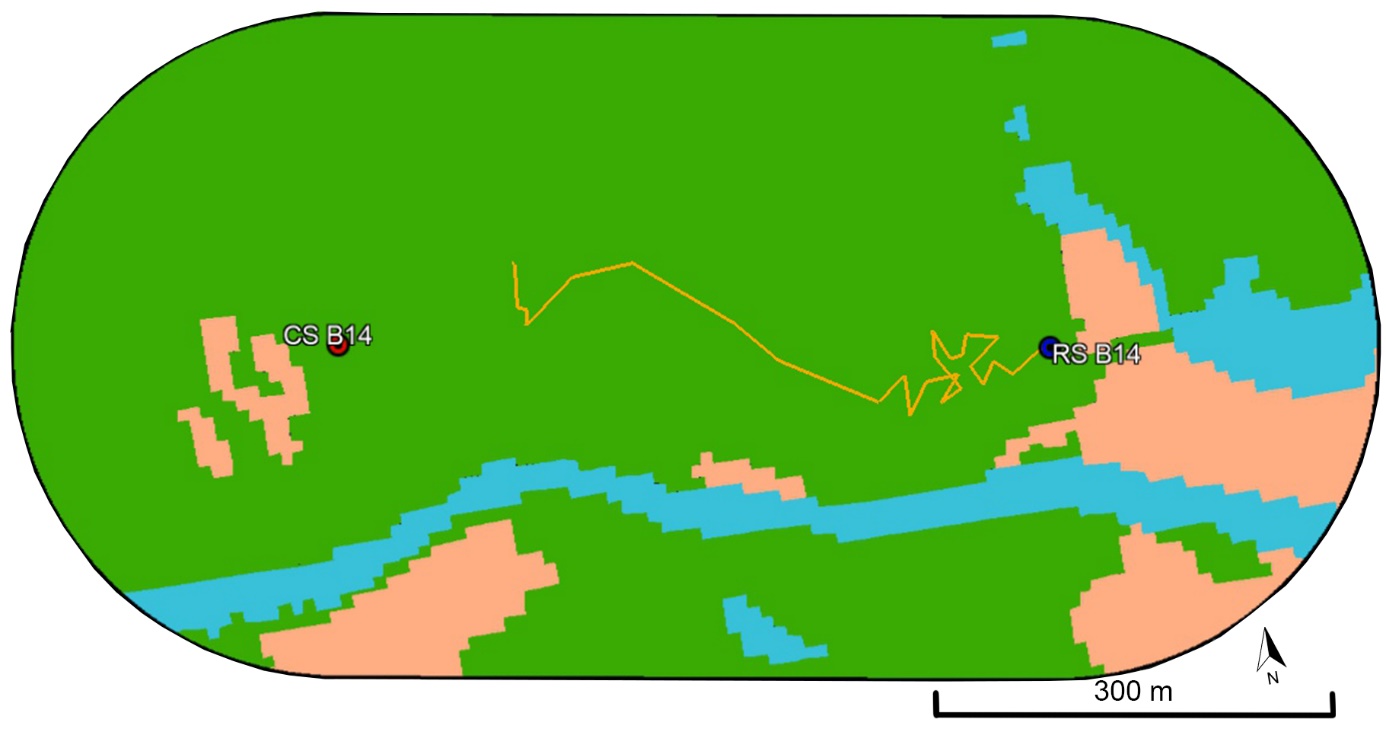


Figure S 2.7: Moved path of B14 individual in Yagirala Forest Reserve


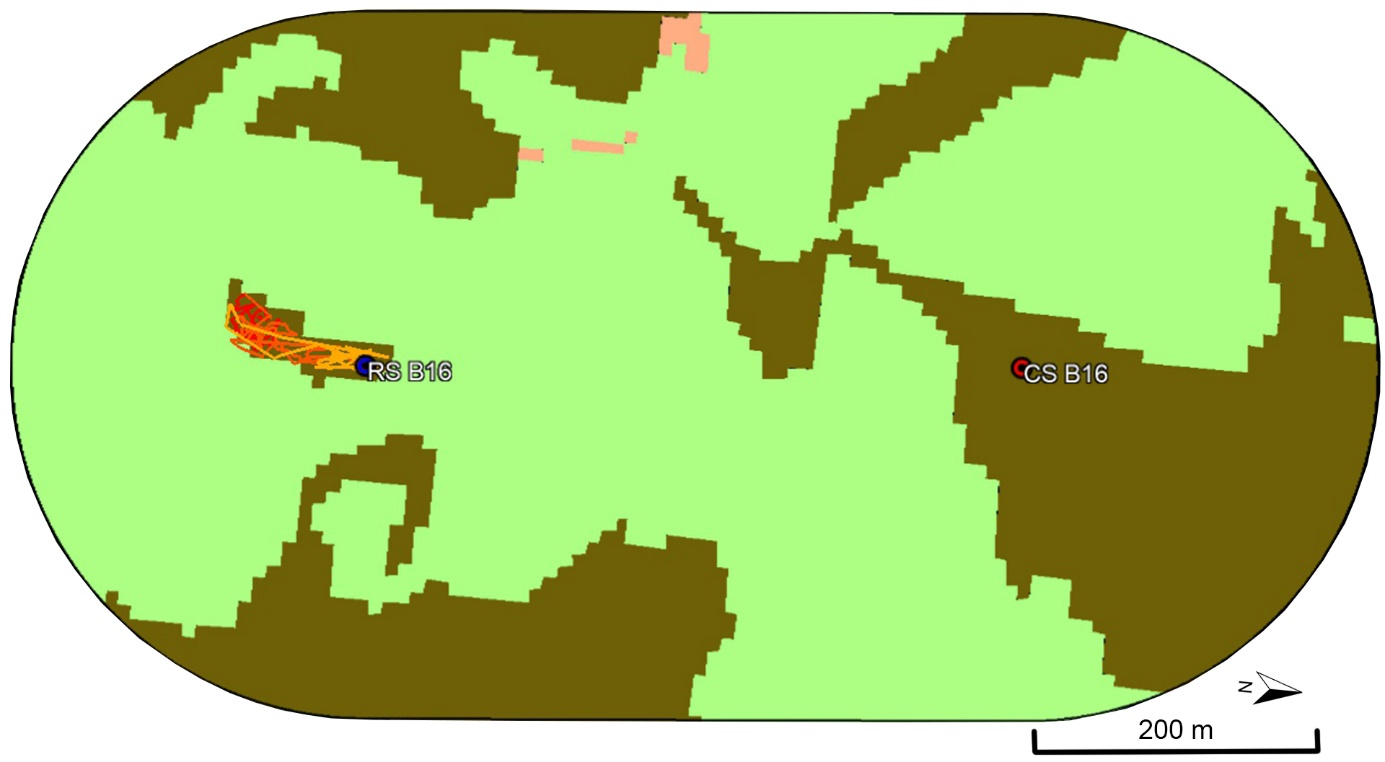


Figure S 2.8: Moved path of B16 individual in Veeoya


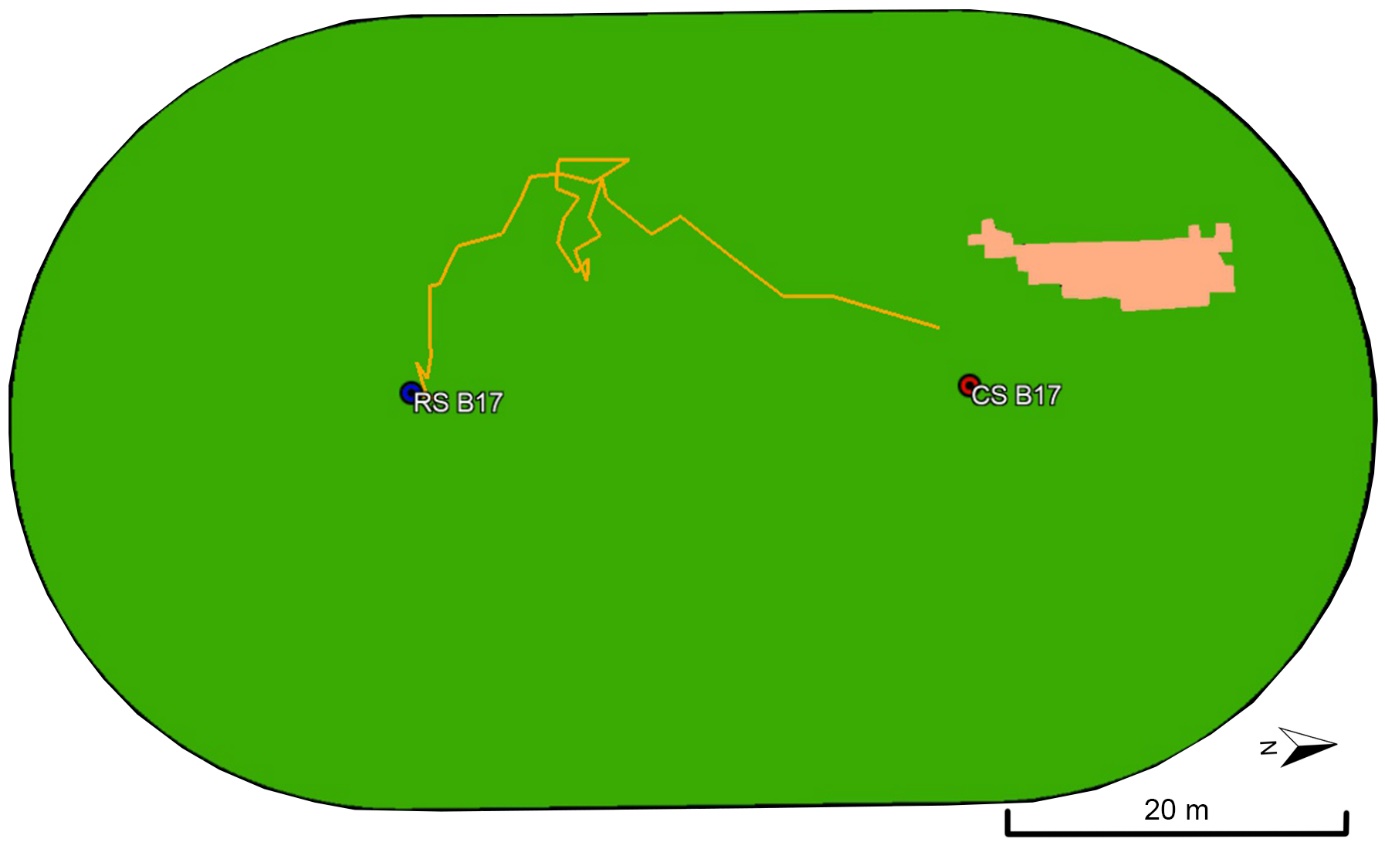


Figure S 2.9: Moved path of B17 individual in Kithulgala Forest Reserve

**Moved trajectories of translocated Tickell’s Blues Flycatcher in both undisturbed and disturbed habitats**


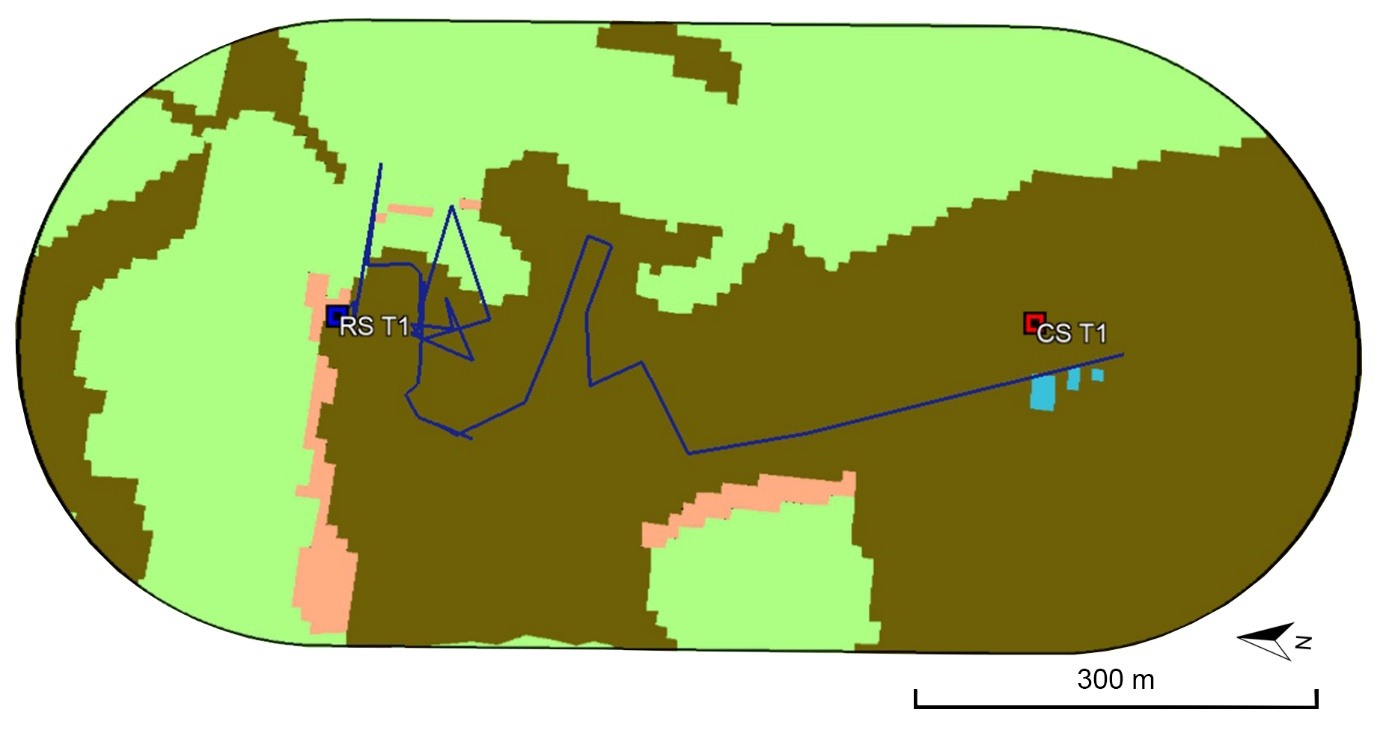


Figure S 2.10: Moved path of T1 individual in Veeoya


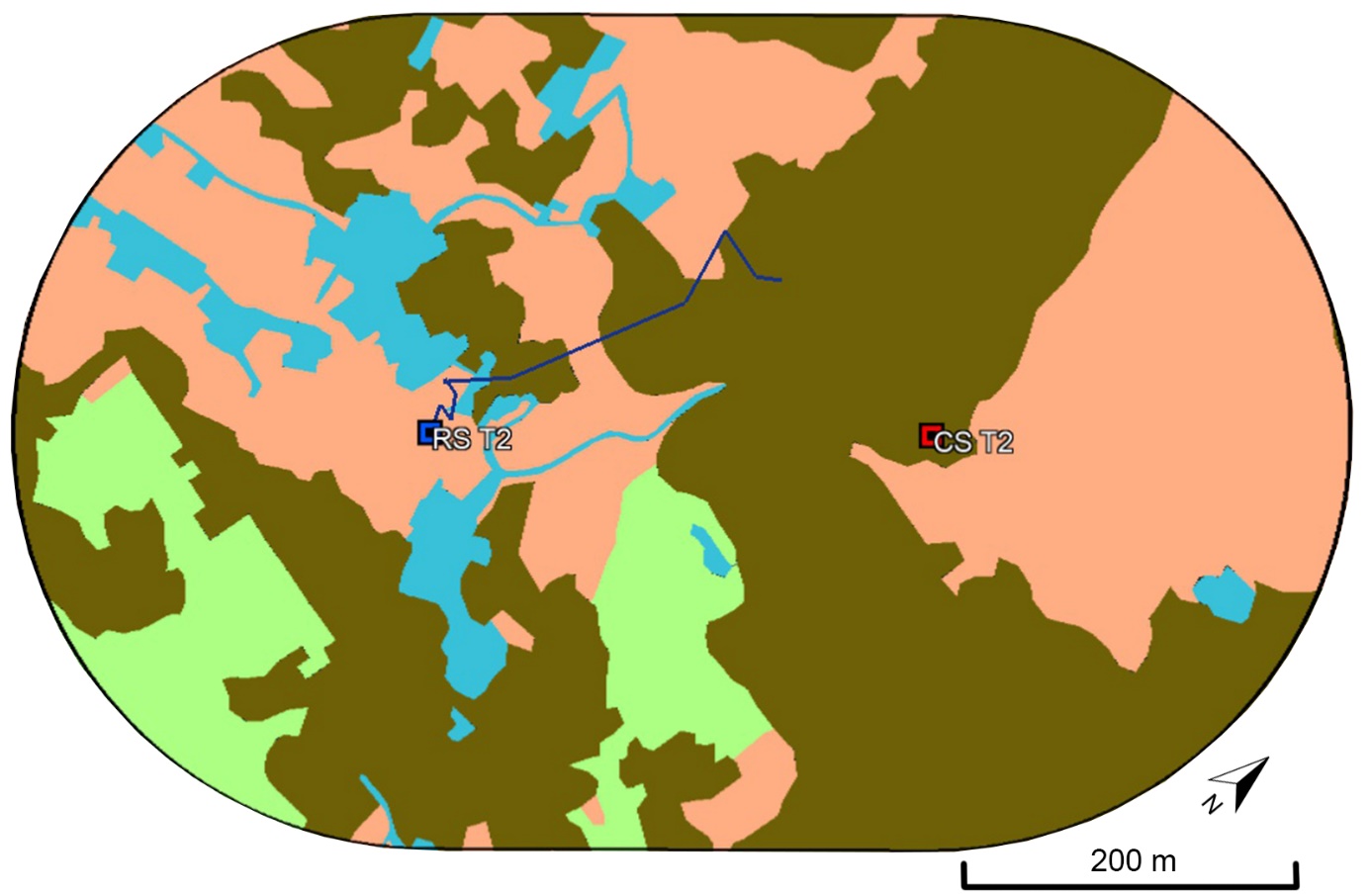


Figure S 2.11: Moved path of T2 individual in Halgolla


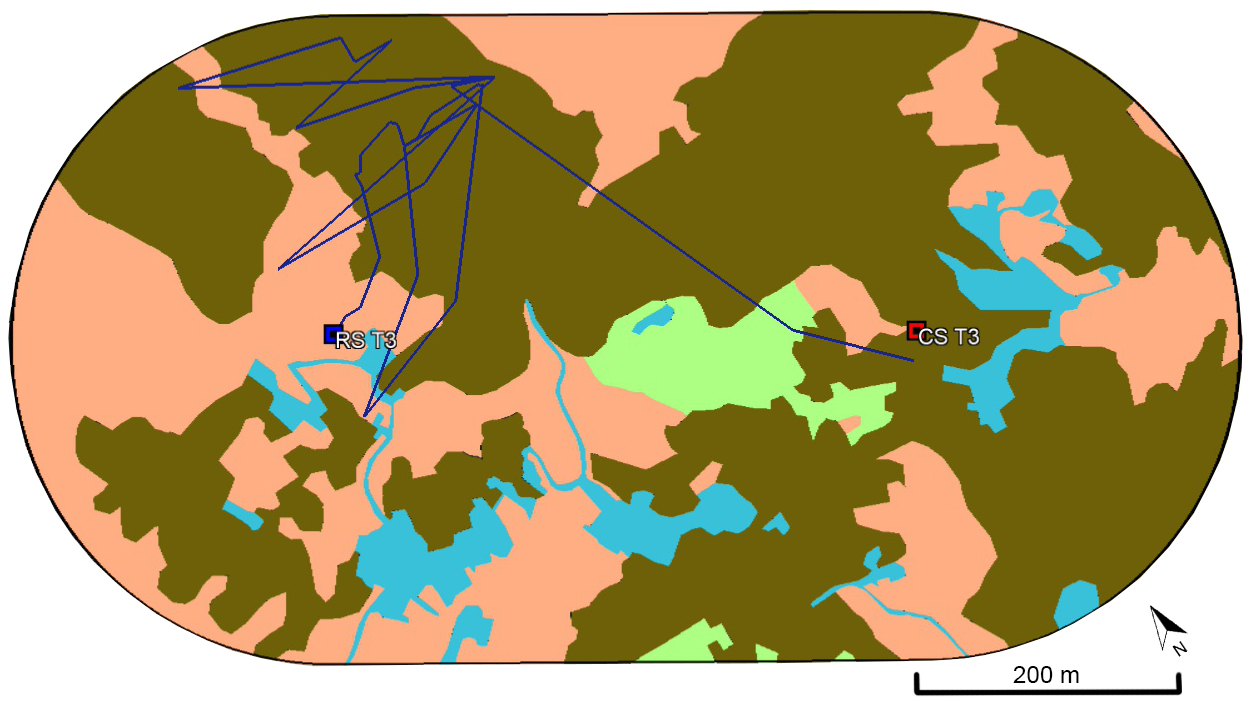


Figure S 2.12: Moved path of T3 individual in Halgolla


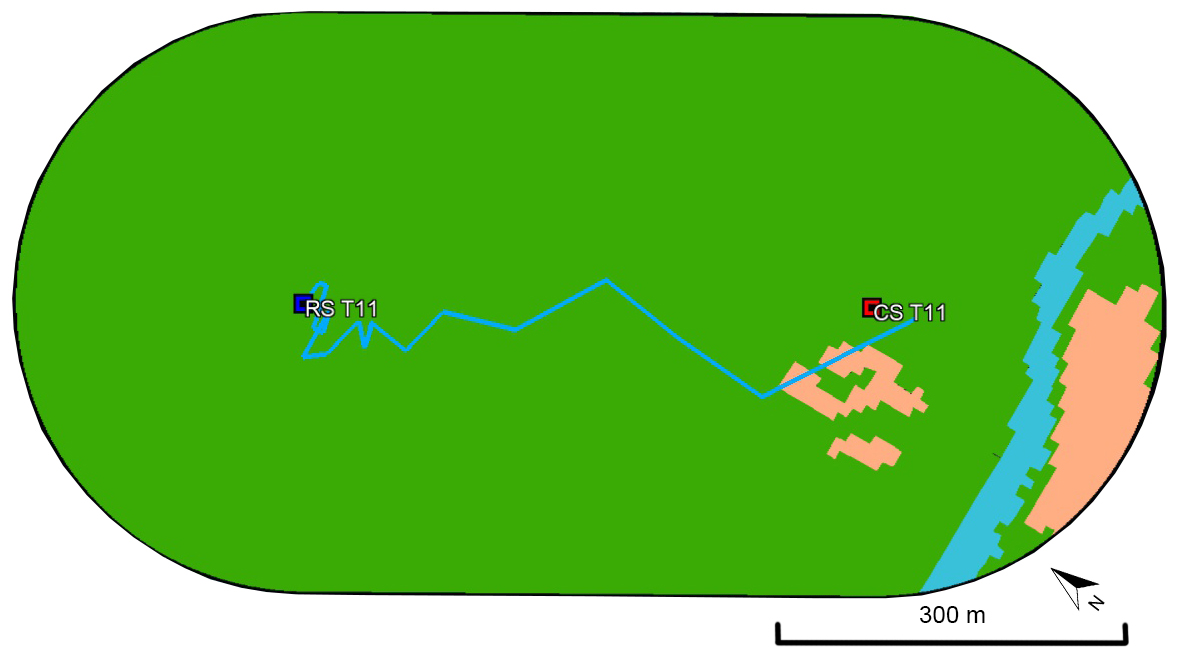


Figure S 2.13: Moved path of T11 individual in Veeoya


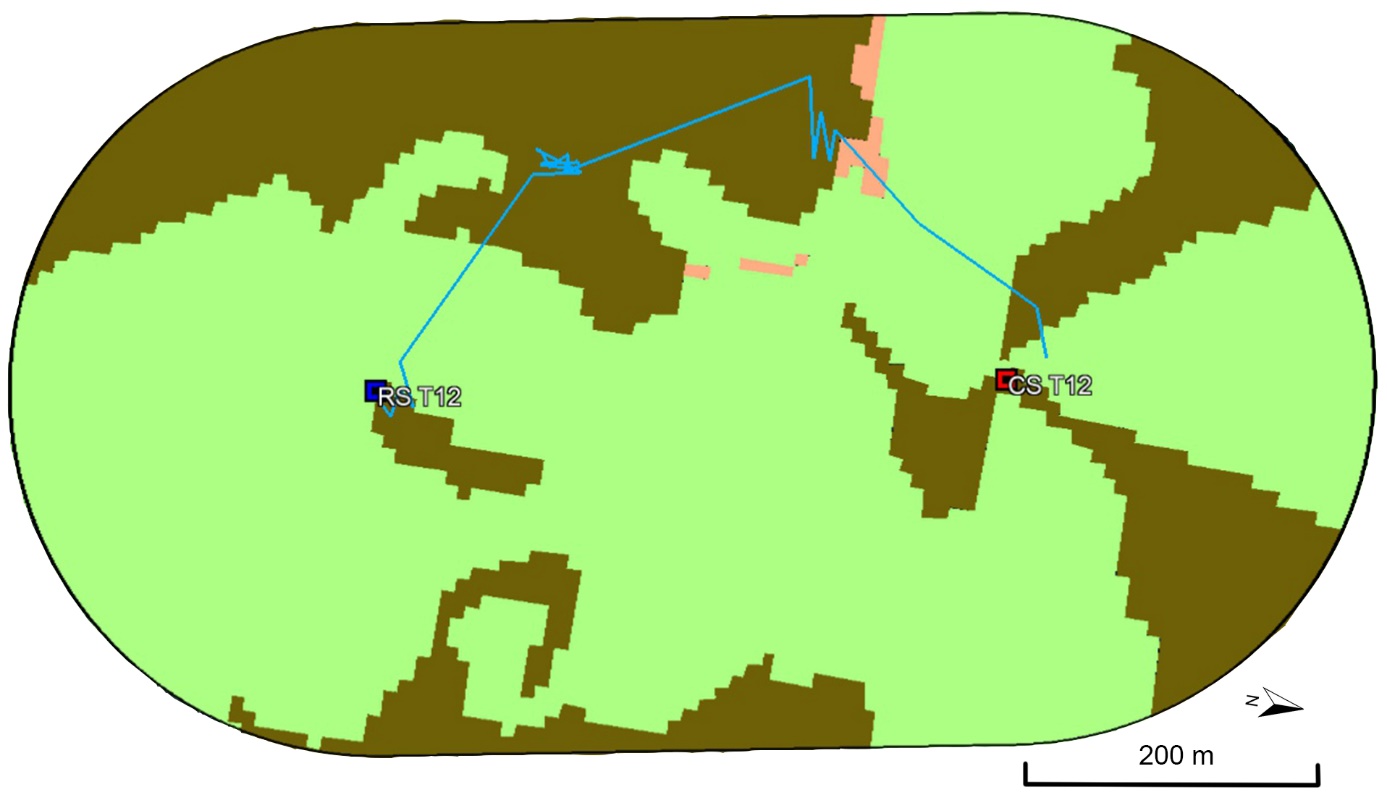


Figure S 2.14: Moved path of T12 individual in Veeoya


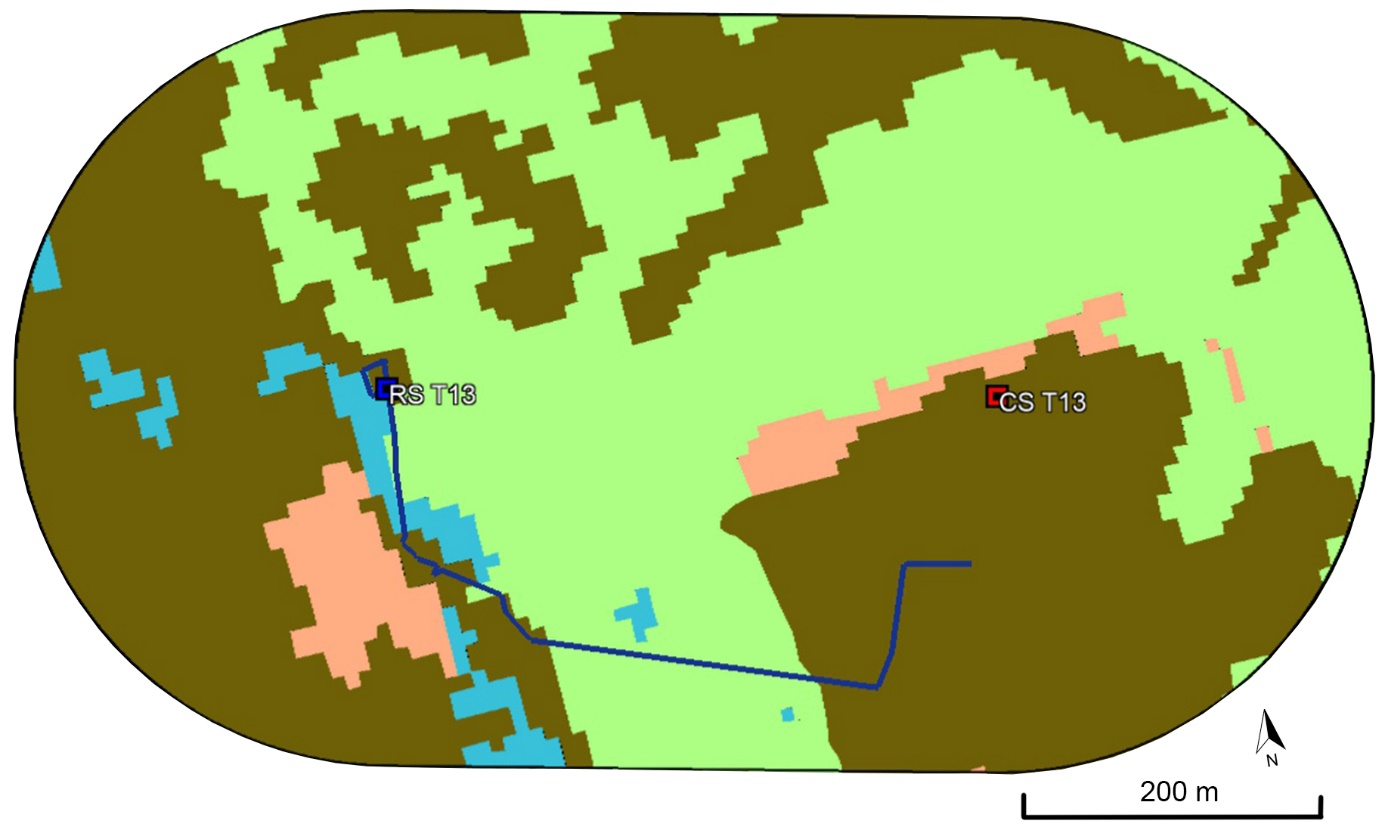


Figure S 2.15: Moved path of T13 individual in Veeoya


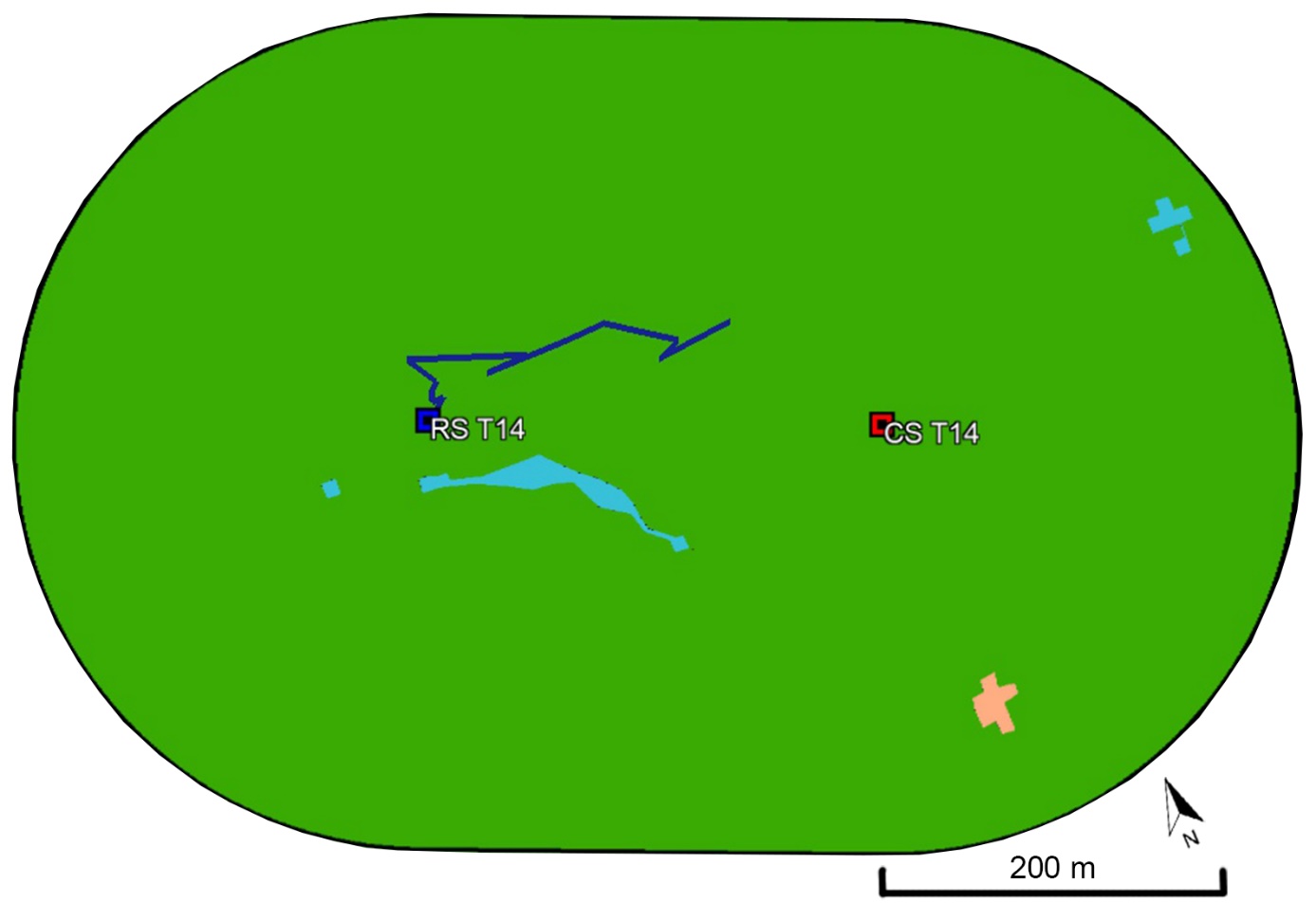


Figure S 2.16: Moved path of T14 individual in Kithulgala Forest Reserve


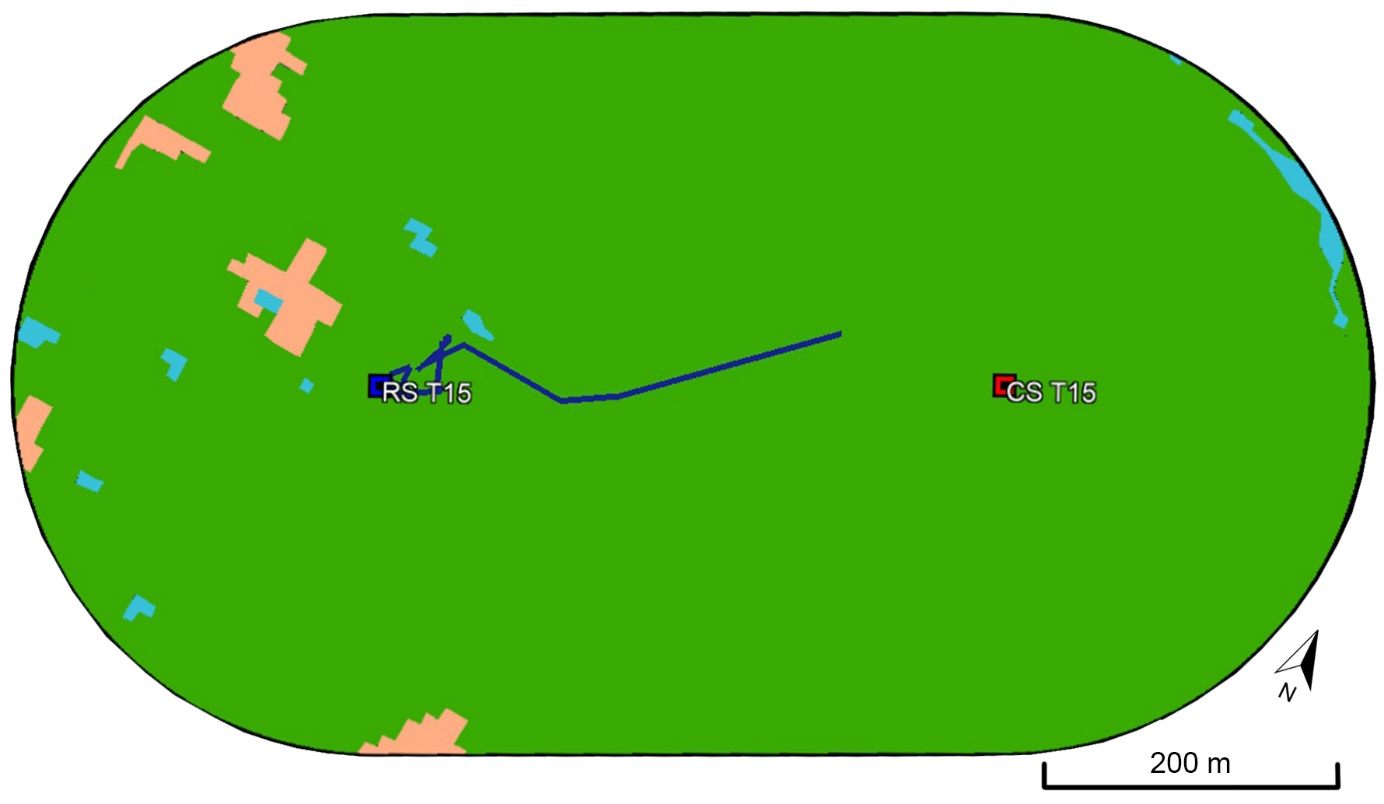


Figure S 2.17: Moved path of T15 individual in Kithulgala Forest Reserve


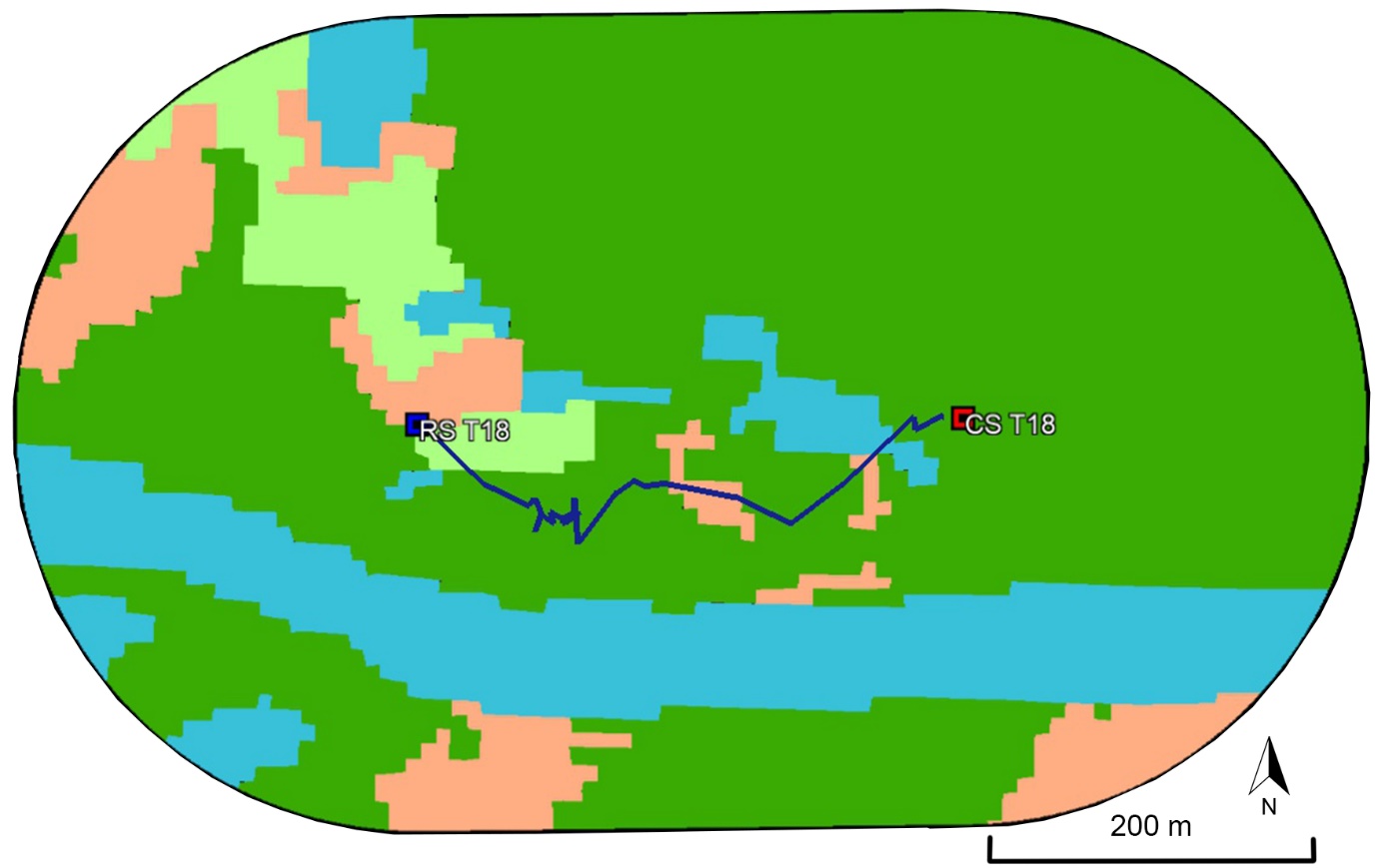


Figure S 2.18: Moved path of T18 individual in Yagirala Forest Reserve


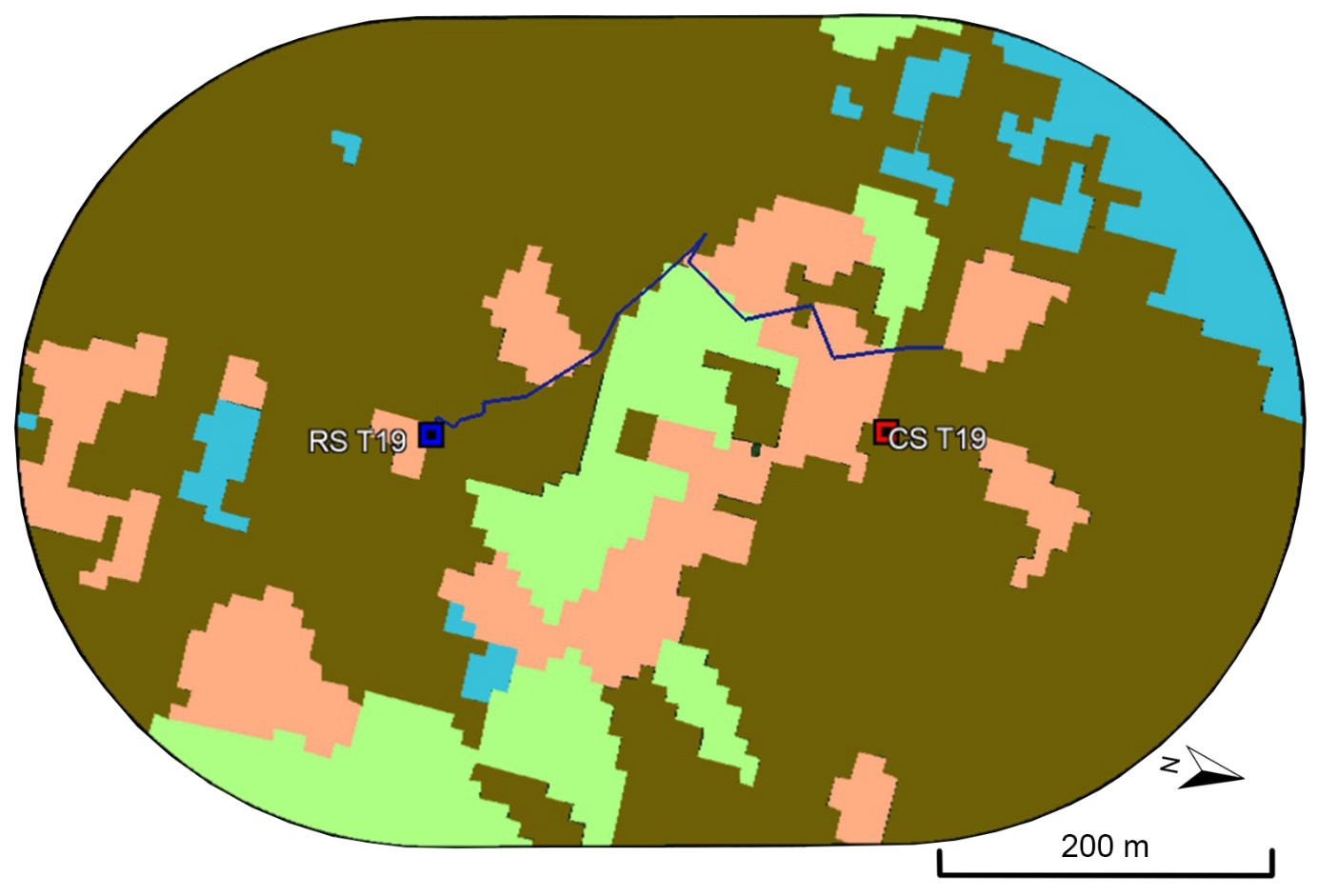


Figure S 2.19: Moved path of T19 individual in Kudaligama

**References for Supplemental**

Billerman, S. M., B. K. Keeney, P. G. Rodewald, and T. S. Schulenberg. 2022. Birds of the World. Cornell Laboratory of Ornithology, Ithaca, NY, USA (<https://birdsoftheworld.org>).

Kotagama, S. W., and G. Ratnavira. 2017. Birds of Sri Lanka: An illustrated guide to the birds of Sri Lanka. Field Ornithology Group of Sri Lanka, Colombo, Sri Lanka.
